# Speciation and adaptive evolution reshape antioxidant enzymatic system diversity across the phylum Nematoda

**DOI:** 10.1186/s12915-020-00896-z

**Published:** 2020-11-26

**Authors:** Lian Xu, Jian Yang, Meng Xu, Dai Shan, Zhongdao Wu, Dongjuan Yuan

**Affiliations:** 1 Key Laboratory of Neuroregeneration, Ministry of Education and Jiangsu Province, Co-innovation Center of Neuroregeneration, Nantong University, Nantong, 226001 China; 2 Department of Parasitology, Zhongshan School of Medicine, Sun Yat-sen University, Guangzhou, 510080 China; 3 Department of Ecology, Jinan University, Guangzhou, 510632 China; 4 BGI Genomics, BGI-Shenzhen, Shenzhen, 518083 China; 5 College of Veterinary Medicine, South China Agricultural University, Guangzhou, 510642 China

**Keywords:** Antioxidant enzyme, Extracellular superoxide dismutase, Gene family evolution, Nematoda, Transcriptome

## Abstract

**Background:**

Nematodes have evolved to survive in diverse ecological niches and can be a serious burden on agricultural economy, veterinary medicine, and public health. Antioxidant enzymes in parasitic nematodes play a critical role in defending against host oxidative stress. However, the features of the evolution of antioxidant enzymes in the phylum Nematoda remain elusive.

**Results:**

Here, we systematically investigated the evolution and gene expression of antioxidant enzymes in the genomes of 59 nematodes and transcriptomes of 20 nematodes. Catalase has been independently lost in several orders, suggesting that it is unnecessary for some nematodes. Unlike in mammals, phospholipid hydroperoxide glutathione peroxidase is widely distributed in nematodes, among which it has evolved independently. We found that superoxide dismutase (SOD) has been present throughout nematode evolutionary process, and the extracellular isoform (SOD3) is diverged from the corresponding enzyme in mammals and has undergone duplication and differentiation in several nematodes. Moreover, the evolution of intracellular and extracellular SOD isoforms in filaria strongly indicates that extracellular SOD3 originated from intracellular SOD1 and underwent rapid evolution to form the diversity of extracellular SOD3. We identify a novel putative metal-independent extracellular SOD presenting independently in *Steinernema* and Strongyloididae lineage that featured a high expression level in *Strongyloides* larvae. Sequence divergence of SOD3 between parasitic nematodes and their closest free-living nematode, the specifically high expression in the parasitic female stage, and presence in excretory-secretory proteome of *Strongyloides* suggest that SOD3 may be related with parasitism.

**Conclusions:**

This study advances our understanding of the complex evolution of antioxidant enzymes across Nematoda and provides targets for controlling parasitic nematode diseases.

## Background

To date, 25% of the ~ 23,000 nematodes have been described to parasitize in animals and plants [1]. Nematodes are aerobic and rely on the oxygen in the atmosphere to metabolize and obtain the energy necessary for life. Low physiological concentrations of reactive oxygen species (ROS) in aerobic organisms are beneficial and involve a series of physiological activities that regulate cell differentiation, proliferation, transformation, apoptosis, protection from invading pathogens, and lifespan [2]. However, an unbalanced elevation of ROS concentration can cause damage to various biological macromolecules, such as carbohydrates, lipids, proteins, and DNA. This in turn can contribute to the development of various diseases, such as cancer, hypertension, diabetes, atherosclerosis, inflammation, and premature aging [3]. Species in the phylum Nematoda are generally large and have successfully adapted to nearly every ecosystem on Earth, from marine to fresh water and soil, from the polar region to the tropics, and from plants to animals [1]. The successful evolution of nematodes requires an effective and flexible defense system against ROS from themselves and the environment.

**Fig. 1:**
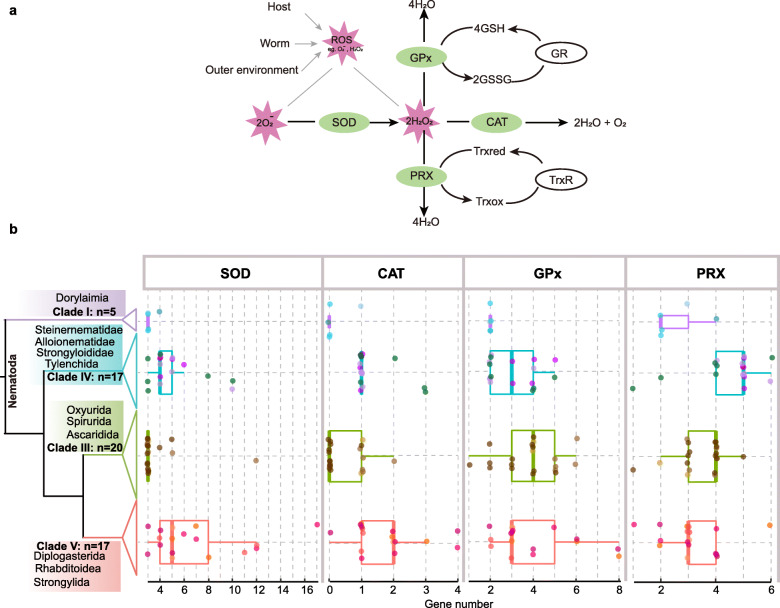
Comparison of gene numbers for antioxidant enzymes in different clades of the phylum Nematoda. **a** Scheme of the antioxidant enzymatic system in a parasite. **b** Gene numbers for SOD, CAT, GPx, and PRX in four clades of nematodes: *n* refers to the species number of the clades studied here. Boxplots show the gene number distribution for antioxidant enzymes in each clade. The purple boxes represent species in Clade I, blue boxes represent species in Clade IV, green boxes represent species in Clade III, and orange represents species in Clade V. The colors of the jitter points indicate the subclades depicted in Fig. 2

Nematodes have developed a complex antioxidant defense system for surviving in the environment or inside their hosts [4]. The typical antioxidant system maintains a dynamic balance in the production and decomposition of ROS in organisms through the synergistic action of superoxide dismutase (SOD), catalase (CAT), glutathione peroxidase (GPx), and peroxiredoxin (PRX) (Fig. 1a). SOD catalyzes the dismutation (or partitioning) of O_2_^−^ into either O_2_ or hydrogen peroxide (H_2_O_2_). The other antioxidant enzymes, CAT, GPx, and PRX, are the major H_2_O_2_-detoxifying enzymes in organisms. CAT, a kind of terminal oxidase, is found in nearly all living organisms exposed to oxygen [5]. GPx functions to promote the decomposition of hydroperoxide, using glutathione as substrate, and reduces its harmful effects on the body. Loss or decrease in GPx activities in organisms can lead to a range of diseases, including diabetes, Keshan disease, and cardiovascular disease [6–8]. The deletion of phospholipid hydroperoxide glutathione peroxidase (PHGPx) causes accelerated aging and a shortened lifespan in *Caenorhabditis elegans* [9]. PRX is a ubiquitous family of antioxidant enzymes that controls cytokine-induced peroxide levels, and thereby mediating signal transduction in mammalian cells. The antioxidant enzymes in parasites are believed to protect them from the ROS that arise from the infection-stimulated host phagocytes [4]. However, the characterization of this antioxidant system in nematodes and its evolutionary adaption to parasitic lifestyle are not well investigated.

An increasing number of genomes of free-living and parasitic nematodes are being sequenced [10], particularly those of three major soil-transmitted nematodes, ascaris [10–13], hookworms [10, 14, 15], and whipworms [16, 17]; the vector-borne nematodes *Anisakis simplex* [10] (herring worm), filarial worms [10, 18–21], *Angiostrongylus* [10, 22], and *Dracunculus medinensis* [10] (guinea worm); and the parthenogenetic parasite *Strongyloides* [23]. Extensive genomic and transcriptomic data have allowed us to explore the growth, reproduction, metabolism, and parasitism of parasites, but this has had far-reaching guiding significance for the prevention and control of parasitic diseases that can seriously harm human and animal health and economic crop growth. In this study, we focused on the genomes of 59 nematode species [10–35] from within four clades (Clades I, III, IV, and V), including free-living, and plant-parasitic, animal parasitic, and entomopathogenic nematodes. We analyzed the gene number, structure, evolution, and expression pattern of antioxidant enzymes in these nematodes, using comparative genomic and transcriptomic approaches during nematode development in free-living and parasitic nematodes in resisting oxidative damage to the worms.

## Results

### Varied gene numbers of antioxidant enzymes in distinct clades of Nematoda

**Fig. 2:**
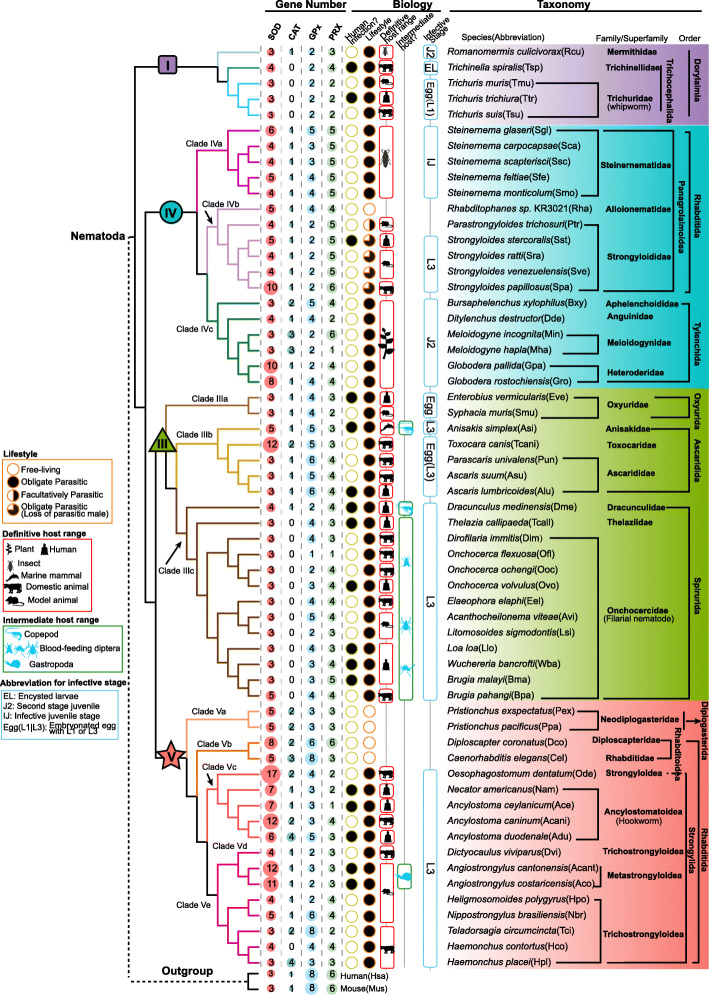
Comparison of the compositions of antioxidant enzymes in 59 nematodes. The sizes of the circles represent the number of antioxidant enzymes in a category. The biology of lifestyle, human infection, definitive host range, and intermediate host range of the nematode are shown. Taxonomic classification is retrieved from the Taxonomy database. The topology of nematode phylogeny is inferred by combining previous studies [10, 24, 36]. The designated shapes for clades and colors for subclades are used consistently throughout the study

A significantly smaller repertoire of antioxidant enzymes was identified in nematodes from Clade I than from other clades (*P* value < 0.05, Wilcoxon rank-sum test, Additional file 1: Fig. S1). GPx and PRX varied within Clades III, IV, and V, and SOD showed the largest variation in Clade V (Fig. 1b). To understand gene number variation during nematode evolution, we inferred the phylogeny of nematodes from previous studies [10, 24, 36] and collected parasitic characters (Fig. 2). CAT was independently lost in Clade I (except the mosquito parasite, *Romanomermis culicivrax*) and IIIc (except the *D. medinensis*, Fig. 2). Nematodes had a variable gene number of SOD, and less GPx and PRX than had been found in mammals (Fig. 2). An obvious expansion of SOD could be found in some species from Clade III (e.g., *Toxocara canis*), Clade IV (e.g., *Strongyloides papillosus*), Clade Vc (e.g., *Oesophagostomum dentatum*), and some genera (cyst nematode *Globodera* in Clade IV and snail-borne nematode *Angiostrongylus* in Clade V) (Fig. 2). Antioxidant enzymes, except for CAT, are multigene families. To further investigate the classification and evolution of antioxidant enzymes, we performed evolutionary analyses for each antioxidant enzyme.

### Overview of SOD gene family evolution

SOD is the only known class of enzyme that is able to autonomously eliminate superoxide anion. Animal SOD includes three isoenzymes according to their subcellular locations, namely, mitochondrial Mn-containing SOD (MnSOD or SOD2), intracellular Cu/Zn SOD (SOD1), and extracellular Cu/Zn SOD (SOD3 or EC-SOD). Phylogenetic analyses of the SOD gene family showed three major clusters (Additional file 1: Fig. S2). To exclude a singleton that may be caused by an annotation issue, we used OrthoMCL to infer the orthologous relationship first. Nematode and mammalian SOD were clustered into 11 groups. We found that most nematode SOD1 were clustered with mammalian SOD1 into one group, except for some SOD1 from the cyst plant-parasitic nematodes (*Globodera*). Nematode SOD2 were clustered with mammalian SOD2 into a single group. However, human and mouse SOD3 clustered together into a single group, while SOD3 from nematodes were grouped into several groups. The orthologous relationship of SOD in nematodes and mammals suggested the conservation of SOD1 and SOD2 and the divergence of SOD3 which was also supported by analyzing the phylogenetic relationship of Cu/Zn SOD from nematodes, insects, mollusks, and vertebrates (Additional file 1: Fig. S3). To study the evolution of SOD gene family in nematodes, we performed phylogenetic analyses for each class.

**Fig. 3:**
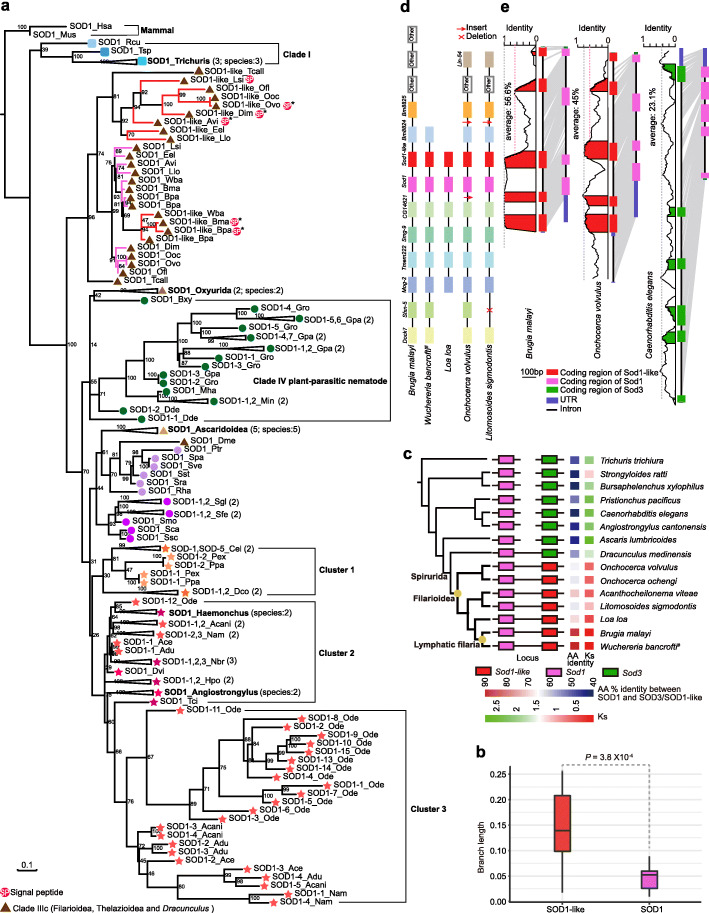
Phylogenetic analyses of SOD1 or SOD1-like proteins from nematodes and mammals, and neighboring and conserved intra- and extracellular SOD isoforms in filariae. **a** Phylogeny of the SOD1 group in nematodes, human, and mouse. The predicted signal peptide of SOD1-like in filariae is indicated with a circled 'SP' and experimental evidence from the literature is indicated by a star. The red branch indicates SOD1-like in filaria, and the pink branch indicates SOD1 in filaria. See Additional file 1: Fig. S5 for a more detailed phylogeny without compressed nodes. The assigned shape and color indicate clades and subclades, respectively. **b** The branch length of SOD1 and SOD1-like in filaria (pink and red branches in panel **a**, respectively). Each genus contains multiple species, and the branch length of a gene in each species is added as their last common node. *P* values were calculated using Student’s *t* test. **c** Neighboring in position and high amino acid identity between *Sod1* and *Sod1*-like in the filariae. AA represents amino acids, and Ks is the synonymous mutation rate. A line connecting two genes indicated that they were neighboring in position. **d** Conserved gene loci around *Sod1* and *Sod1*-like in Filarioidea with gene number more than six in the scaffold. **e** Conservation of *Sod1* and *Sod1*-like in structure and genomic sequences. Genomic identity was calculated at a 10-bp sliding window based on pairwise alignment

In phylogenetic analyses of 61 SOD2 sequences of nematodes, SOD2 from species in Clades I, III, and V clustered together, but SOD2 from species in Clades IVa (insect-parasitic nematodes), IVb, and IVc (plant-parasitic nematodes) were not (Additional file 1: Fig. S4). Phylogenetic analyses of the SOD1 group (Fig. 3a and Additional file 1: Fig. S5) showed that some underwent extensive lineage- (plant cyst nematode *Globodera* [31]) and species-specific expansion (nodule nematode *Oesophagostomum dentatum*). SOD1 from nematodes in Clade III were clustered together within subclades, except for SOD1 in the guinea worm *D. medinensis*, but they diverged within clades. Two “isoforms” (details discussed below) were observed in filaria and *Thelazia callipaeda* (oriental eyeworm), but one copy was observed in other Clade III nematodes. In Clade V, SOD1 from free-living nematodes (*C. elegans*, *D. coronatus*, and *Pristionchus*) clustered together (cluster 1 in Fig. 3a), and SOD1 from parasitic nematodes clustered into two groups (clusters 2 and 3 in Fig. 3a). SOD1 in cluster 3 were Clade Vc-specific and underwent extensive expansion in *O. dentatum* (Fig. 3a and Additional file 1: Fig. S5).

**Fig. 4:**
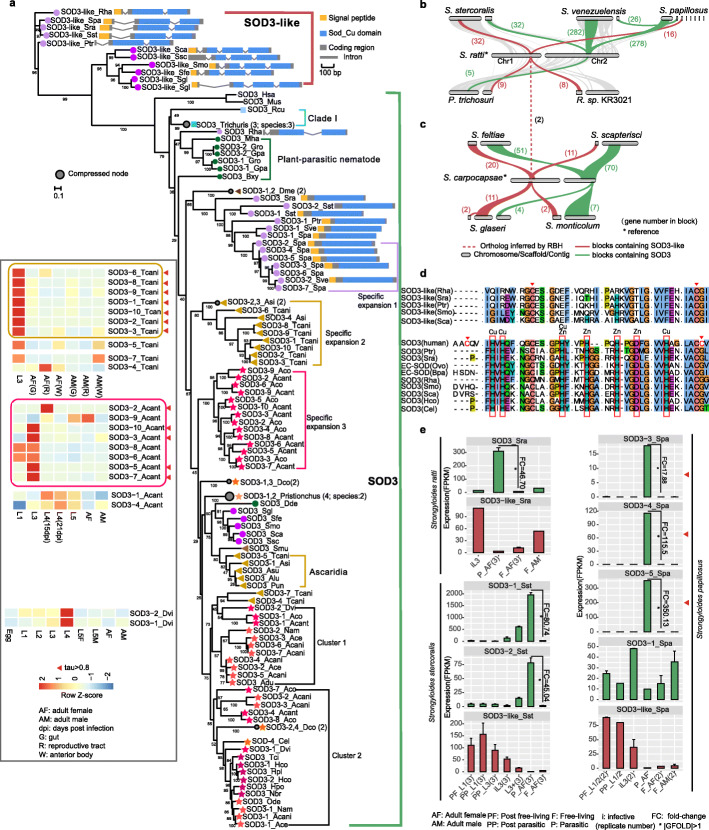
Evolution of SOD3 and SOD3-like genes in nematodes, human, and mouse. **a** Phylogeny of SOD3 and SOD3-like genes and gene expression of SOD3 across developmental stages in three nematodes with available RNA-seq data. Lineage-specific expansion in the genus *Angiostrongylus*, *T. canis*, and *S. papillosus* was highlighted. The gene structures of SOD3-like in Clade IVa and Clade IVb, and SOD3 in Clade IVb are shown. Shapes and colors indicate different clades and subclades, as depicted in Fig. 2. Bootstrap values are shown in the node. Scale bar represents the number of amino acid substitutions per site. **b**, **c** Syntenic blocks in chromosomes or scaffolds or contigs containing SOD3 or SOD3-like in species from Clade IVa and Clade IVb. Numbers in the parentheses are gene number in blocks. **d** Fragments of multiple sequence alignments of EC-SOD and SOD3-like. Metal-binding sites are shown in the red box. Red triangles show disulfide bonds. Dots are used to separate blocks. Full alignment is shown in Additional file 1: Fig. S11. **e** Gene expression patterns of SOD3 or SOD3-like genes in *Strongyloides ratti* (Sra), *S. stercoralis* (Sst), and *S. papillosus* (Spa). Genes with row maximum expression (FPKM) less than 10 were not shown. Detailed expression values were shown in Additional file 6: Table S6. FPKM less than 1 was set 1 to calculate fold change. GFOLD value was calculated between free-living adult female (F_AF) and parasitic adult female (P_AF)

Phylogenetic analyses of the SOD3 group showed three major clusters, namely, nematode SOD3, mammalian SOD3, and SOD3-like (illustrated below) from nematode Clades IVa and IVb (Fig. 4a and Additional file 1: Fig. S7). The lineage-specific expansion of SOD3 was observed in the genus *Angiostrongylus* and in *T. canis*. SOD3 from *T. canis* and *A. simplex* were divided into two and three branches, respectively. In one branch, SOD3 from *T. canis* and *A. simplex* were clustered with SOD3 from three other species of Ascaridida, suggesting that SOD3 from this branch were ancient. In the second branch (specific expansion 2 in Fig. 4a), SOD3 from *T. canis* and *A. simplex* were clustered into a separated branch. Considering the phylogenetic relationship and different life cycles between *T. canis* and *A. simplex*, the SOD3 of specific expansion 2 in these two species showed possibly independent duplication and divergence. SOD3 in the genus *Angiostrongylus* underwent extensive expansion (9–10) in their last common ancestor and were divided into three clusters, one clustered with SOD3 from other Clade V nematodes (cluster 2 in Fig. 4a), one clustered with hookworm (cluster 1 in Fig. 4a), and the last being lineage-specific (specific expansion 3 in Fig. 4a). Two clusters of SOD3 were also observed in hookworms and bovine lungworm *Dictyocaulus viviparus* (Fig. 4a). Available public RNA-seq datasets [13, 22] showed expression specificity (tissue specificity index *τ* ≥ 0.8) at a certain stage of most lineage-expansion of SOD3 isoforms in *T. canis* (seven out of seven) and *A. cantonensis* (five out of eight) (Fig. 4a, Additional file 6: Table S6).

#### SOD3 possibly originated from SOD1, supported by the presence of extracellular SOD1-like in filaria

Interestingly, we found seven SODs with signal peptide clustered with intracellular isoform in filaria (Fig. 3a), five of which were confirmed with experimental evidence [37–39]. It should be admitted that typical SOD3 is extracellular, with signal peptide, and typical SOD1 is intracellular, without signal peptide. We called the branch that contained these seven SOD1 genes with signal peptide as extracellular SOD1-like. We also found SOD1 and SOD1-like instances in lymphatic filaria clustered together with SOD1 from other filariae, such as *Onchocerca volvulus* (Fig. 3a). Further, the branch length of the SOD1-like group was significantly longer than the SOD1 group (Student’s *t* test, *P* = 3.8 × 10^− 4^, Fig. 3b), suggesting a higher rate of protein evolution. We found that SOD1 and SOD1-like were neighboring in gene position in the order Spirurida, but in other nematodes, including well-studied *C. elegans*, intra- and extracellular SOD were not, even distributed in different scaffolds or chromosomes (Fig. 3c**,** the position of intra- and extracellular SOD loci in additional nematodes is shown in Additional file 1: Fig. S6). Further, the identity of amino acids found in SOD1 and SOD1-like was the highest (89–92%) in lymphatic filaria, 63–71% in other filariae, and only 41–55% in other nematodes. In addition, low Ks (synonymous substitutions per synonymous site) values (0.07–0.65, except for rodent filaria *Litomosoides sigmodontis*) between intra- and extracellular SOD1 (Fig. 3c) suggested that these genes had recently duplicated and diverged.

We also examined the synteny of chromosomes or scaffolds containing SOD1 and SOD1-like in filaria (gene number in a scaffold more than six genes were considered; information of fragment scaffolds/contigs encoding SOD1 or SOD1-like was listed in Additional file 1: Table S1). We found that this was conserved upstream of SOD1 but not downstream of extracellular SOD1-like (Fig. 3d). Then, we evaluated the genomic conservation of SOD1 and SOD1-like. The average identity of SOD1 and SOD1-like in filaria (45–55.6%) was higher than SOD1 and SOD3 in *C. elegans* (23.1%, Fig. 3e). Specifically, almost identical regions were found at the 3′ end of the SOD1 and SOD1-like in *B. malayi*, and a high identity (about 80%) was shown in three coding regions of the SOD1 and SOD1-like 3′ end in *O. volvulus*. The identity of SOD1 and SOD1-like 5′ end was similar (around 60%) in *B. malayi* and *O. volvulus*. However, there was only at most 40% identity throughout the whole region of SOD1 and SOD3 in *C. elegans*, and no obvious difference was found between the coding and noncoding regions or the 5′ end and 3′ end (Fig. 3e). This result strengthened the possibility that the extracellular isoform originated from the intracellular isoform by duplication and underwent genetic variation first at the 5′ end, and then at the noncoding region of the 3′ end, and last throughout the whole region, forming obviously divergent intra- and extracellular isoforms in the nematodes.

#### SOD3 is possibly related with parasitism in *Strongyloides*

SOD3 in species from Clade IVb showed sequence divergence and varied in gene number between free-living *Rhabditophanes* and parasitic Strongyloididae. SOD3 of *S. papillosus* underwent extensive expansion (seven copies) and duplication (two copies) in *S. stercoralis* (Fig. 4a). Synteny analyses showed that gene order in the syntenic block containing SOD3 was highly conserved in *Strongyloides*, with small-scale synteny between *S. ratti* and *P. trichosuri*, but lack synteny between *S. ratti* and *Rhabditophanes* (Fig. 4b). SOD3 was also identified and conserved in *Steinernema*, but it diverged from SOD3 in Clade IVb (Fig. 4a, c). The average CDS (coding sequence) number (2.49 ± 1.64) of whole protein-coding genes in Clade IVb was significantly less than other clades or subclades (Wilcox test, *P* < 0.001, Additional file 1: Fig. S8). It has been shown that substantial intron losses occurred before the evolution of the *Rhabditophanes*-*Parastrongyloides*-*Strongyloides* clade [23]. SOD3 in Strongyloididae was intronless, suggesting that complete intron loss of SOD3 occurred in the last common ancestor of Strongyloididae, but has three CDSs in free-living *Rhabditophanes* that has close relationship with Strongyloididae (Fig. 4a, Additional file 1: Figs. S9 and S10). These evidences suggested that SOD3 in free-living *Rhabditophanes* and parasitic Strongyloididae have been diverged.

A striking biology of the genus *Strongyloides* is female-only parasitic lifestyle. RNA-seq data, including parasitic adult female (simplify as P_AF) and free-living adult female (simplify as F_AF) stages in public database [23, 40, 41], enable us to deeply investigate potential roles of genes in nematode biology. Analysis of transcriptome data of three *Strongyloides* species showed that SOD3 were coordinately significant upregulation in P_AF compared with F_AF with fold change of 46.7 in *S. ratti*, 45 and 81 in *S. stercoralis* (two copies), and 18~350 in *S. papillosus* (three out of four relatively high expression of SOD3) (|GFOLD| > 1, Fig. 4e). Further, somatic proteomes of parasitic and free-living females of *S. ratti* also show that SOD3 (original gene id: SRAE_2000310200) is significantly upregulated in parasitic females compared with free-living females with fold change of 16.5 [23], which is accordant with the result of transcriptional level. In addition, SOD3 is also detected in excretory-secretory (ES) proteome of *S. ratti* [23, 42], suggesting its importance in parasite-host interaction. Thus, considering sequence divergence of SOD3 between free-living and parasitic nematodes in Clade IVb, and extremely high expression in parasitic female stages, and presence in ES, we propose that at least some copies of SOD3 in *Strongyloides* may be beneficial for its parasitism (details see Additional file 1: Fig. S13).

#### Putative novel metal-independent extracellular SOD isoform (SOD3-like) independently arose in *Steinernema* and Clade IVb

We also found a group of “SOD3” in species from Clade IVb (*Strongyloides* and others) and *Steinernema* that diverged from mammalian and nematode SOD3. Moreover, most of them encoded a signal peptide (Fig. 4a). Thus, we called the genes in this group as SOD3-like. Low amino acid identity (an average of 30%) was observed between SOD3 and SOD3-like. A local synteny of the gene order in genomic region containing SOD3-like was conserved in Clades IVa and IVb, respectively (Fig. 4b, c). No syntenic block was detected between *S. ratti* (Clade IVb) and *S. scapocapsae* (Clade IVa), with the exception of two orthologous pairs (Fig. 4b, c). The branch length of SOD3-like in *Steinernema* was longer than that in *Strongyloides*, suggesting a rapid divergence of SOD3-like in *Steinernema* (Fig. 4a). Thus, we proposed that SOD3-like might independently occur and have undergone rapid evolution in *Steinernema*. The alignment of amino acids for SOD3-like and SOD3 in nematodes and human showed that SOD3-like lacked key metal-binding residues (Fig. 4d and Additional file 1: Fig. S11). A 3D structural model of human and barber’s pole worm (*Haemonchus contortus*) SOD3, and *S. ratti* SOD3 and SOD3-like also showed that SOD3-like could not bind with Cu or Zn (Additional file 1: Fig. S12). Unlike SOD3 in Strongyloididae species, the CDS number in SOD3-like Strongyloididae species was multiple (three CDSs, Additional file 1: Figs. S9 and S10) and with a high identity (amino acid identity 69%) between *Rhabditophanes* and Strongyloididae. The transcriptomes of three *Strongyloides* species showed coordinately high expression of SOD3-like in the larval stages (e.g., L1/2), while low expression in the adult stage (Fig. 4e). This suggests a functional putative metal-independent extracellular SOD in *Strongyloides*, which requires further experiments to investigate its activity and stability.

### Loss, lineage-specific expansion, and duplication of CAT in several nematodes

CAT was independently lost in species from Trichocephalida (Clade I), filaria, and *T. callipaeda* in Clade III, but present in their closest relatives (*R. culicivorax* in Clade I and *D. medinensis* in Clade III). Phylogenic investigation showed that 62 nematode orthologs of CAT were conserved within subclades but diverged within clades (Additional file 1: Fig. S14A). In plant-parasitic nematodes, one CAT of root-knot nematode (*M. hapla*) clustered with CATs of other plant-parasitic nematodes, while the other five CATs of two root-knot nematodes clustered into a single cluster.

To determine whether the single cluster CAT of root-knot nematodes is lineage-specific, we analyzed other plant-parasitic nematodes [43–46], including five other root-knot nematodes. Three to eleven CATs were encoded in the draft genomes of root-knot nematodes. Phylogenetic investigation (Additional file 1: Fig. S14B) supported the contention that the specific CAT occurred and diverged in their last common ancestor. The reproduction mode in root-knot nematode is complex and different from that of other plant-parasitic nematodes, in that some of them show facultative meiotic parthenogenesis (*M. hapla*, *M. graminicola*, and *M. floridensis*), while others are obligatory mitotic parthenogenesis (*M. incognita*, *M. arenaria*, *M. javanica*, and *M. enterolobii*), which leads to aneuploid and polyploid genomes [47]. Approximately 2:1 or more than 3:1 of CAT gene number in root-knot nematodes were observed in mitotic parthenogenesis compared to *M. hapla* (Additional file 1: Fig. S14B). We next detected conserved nematode orthologs among seven root-knot nematode genomes and estimated ortholog number ratio relative to the diploid *M. hapla* (1:1, 2:1, ≥ 3:1, Additional file 1: Fig. S14C and Table S2). The result showed a higher proportion of the duplicated BUSCOs (13.1–36.7%) in four mitotic parthenogenetic species than that in three meiotic parthenogenetic species (0.4–3.0%, Additional file 1: Table S2). Further, summarization of 2:1 or 3:1 of orthologs relative to *M. hapla* showed large (26–42%) in four mitotic parthenogenetic species, especially in *M. arenaria*, while was rare (≤ 5%) in two meiotic parthenogenetic species. Result was accordant with a previous study [48]. Thus, multiple copy number of CAT in each cluster in mitotic parthenogenetic root-knot nematodes possibly was the result of their genomic characterization.

Gene expression of CATs across different development stages in *M. incognita* showed two divergent expression patterns that one (Ctl-1, Ctl-3) was high expression in endophytic stages (L3, L4, and adult female) and the other (Ctl-2) showed high expression in exophytic L2 stage (infective juvenile), while both showed low expression in exophytic egg stage (Additional file 1: Fig. S14D), according to the data of Inchan et al. [49]. Two CATs encoded in pinewood nematode *B. xylophilus* genome showed two divergent patterns according to RNA-seq in developmental stages [50]. One (Ctl-2) showed highly expression in the L2 stage, while the other (Ctl-1) showed wide expression across developmental stages, including egg (Additional file 1: Fig. S14D). CAT could induce high-virulence *B. xylophilus* under H_2_O_2_-induced stress [51]. Lineage-specific expansion and high expression in infective or parasitic stages of CAT may benefit for root-knot nematode colonization.

### Abundant phospholipid hydroperoxide glutathione peroxidase (PHGPx) was a major GPx isoform in nematodes

Mammalian GPx could be divided into selenium-containing proteins (GPx1-4) and nonselenium-containing proteins (GPx5-8) [52]. GPx4, also known as PHGPx, is one of the most abundant isoforms and interferes directly with hydroperoxidation of lipids [53]. In OrthoMCL clustering analyses, GPx separated into three groups, namely mammalian GPx7 and GPx8, nematode PHGPx with mammalian GPx4, and nematode non-PHGPx (termed NPHGPx here) with mammalian GPx1, 2, 3, 5, and 6.

#### Independently duplicated and distinct divergence of PHGPx across nematode clades

**Fig. 5:**
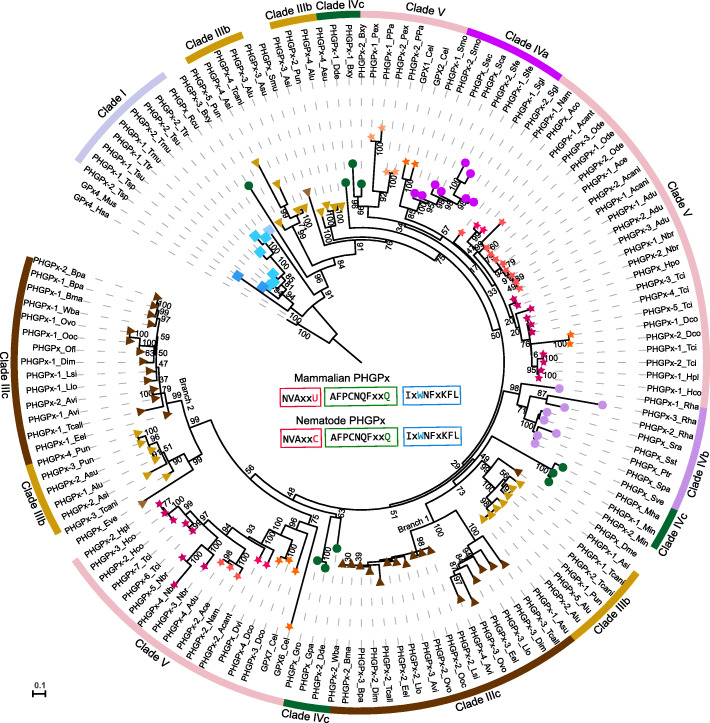
Phylogenetic analyses of nematode and mammalian PHGPx. PHGPx from mammals was used as outgroup. Shape and color leaf decoration indicated clades and subclades, respectively, as depicted in Fig. 2. Scale bar represents the number of amino acid substitutions per site

Two PHGPx have been identified in most nematodes, but only one (GPx4) has been found in mammals (Fig. 5). Two PHGPx in *Trichuris* species might have been formed by tandem duplication in their last common ancestor. The expression pattern of PHGPx in *T. suis* and *T. muris* showed one (PHGPx-2) highly expressed in adult female, while (PHGPx-1) stably expressed across the development stages [16, 17] with a relatively high expression in adult male stage (Additional file 6: Table S6). Recently duplicated PHGPx was also found in the genus *Pristionchus* (Clade V, Fig. 5). Duplicated PHGPx after speciation was also detected in *Steinernema* (Fig. 5). Duplicated and divergent PHGPx was found in nematodes from Clades III and V (Fig. 5). PHGPx in Clade III varied in gene number (1–5) and its sequences diverged into multiple clusters (Fig. 5). Only one PHGPx was found in *D. medinensis*, but two to three in *T. callipaeda*, as well as in 11 filarial worms, dividing into two branches (branches 1 and 2 in Fig. 5). In branch 1, the lymphatic filarial worms had one PHGPx, but other non-lymphatic filarial nematodes had two PHGPx, which were separated into two subbranches. It may be simply annotation issues that PHGPx-3 was absent in the original gene annotation for *O. volvulus*, but it presented clearly in its genome with transcriptional evidence (Additional file 1: Fig. S15). These data suggested that PHGPx, in this branch of non-lymphatic filaria, might have experienced duplication and loss in the last common ancestor of lymphatic filaria. The PHGPx (at least four copies) in species from Ascaridida were divided into four branches: two of them clustered with filariae, suggestive of an origin in their last common ancestor, but two other copies might have experienced independent duplication and then diverged in the ascarid lineage. In Clade V, PHGPx were clustered into two major branches and varied in gene number in some species, except for *Pristionchus* (Fig. 5). PHGPx duplication has continuously proceeded, being basal, intermediary, and recent, across the phylum Nematoda.

#### Loss and pseudogenization of NPHGPx in several nematodes

Mammalian NPHGPx has multiple copies (GPX-1, 2, 3, 5, and 6), while most nematodes have only one copy, with the exception of *Steinemema* and oxyurid nematodes (Additional file 1: Fig. S16). We found that NPHGPx was absent in Trichocephalida (Clade I) but present in *R. culicivorax.* Three NPHGPx from oxyurid nematodes were identified and separated into three branches and tandemly located in the genome, suggesting that NPHGPx were duplicated in their last common ancestor and then diverged. One NPHGPx from the oxyurid nematodes lacks triads of amino acid residues (C, Q, and W, Additional file 1: Fig. S16), which may not act as GPx. It has been shown that *B. malayi* NPHGPx (also known as gp-29) cannot metabolize H_2_O_2_, and *D. immitis* NHGPx can metabolize a limited amount of H_2_O_2_, while *O. volvulus* NPHGPx is a pseudogene with a frameshift in sequence that lacks the catalytic triad of Cys residues [4]. A frameshift also occurred in other two NPHGPx orthologs from *Onchocerca* species, indicating that NPHGPx underwent pseudogenization in these species (Additional file 1: Fig. S16). Thus, although NPHGPx orthologs can be found in these nematodes, their antioxidant function might have been modified. Unlike the relationship between PHGPx from the filaria and from the ascarids, NPHGPx from ascarids did not clustered with that from filarial worms and had high expression in larvae and extremely low expression in adult, as inferred from the transcriptomes of *T. canis* and *A. suum* (Additional file 1: Fig. S16 and Additional file 6: Table S6). In Clade IV, two NPHGPx paralogs in species from *Steinernema* appeared before speciation and diverged into two clusters (Additional file 1: Fig. S16). Expression data showed low expression of NPHGPx-2 in *Steinernema*, which clustered with potential nonfunctional NPHGPx from Oxyurida (Additional file 1: Fig. S16), while NPHGPx-1 was highly expressed in the infective stage and had extremely low expression in the eggs (Additional file 1: Fig. S16).

### Cytosolic Prx1 was the major H_2_O_2_ scavenger in PRX

**Fig. 6:**
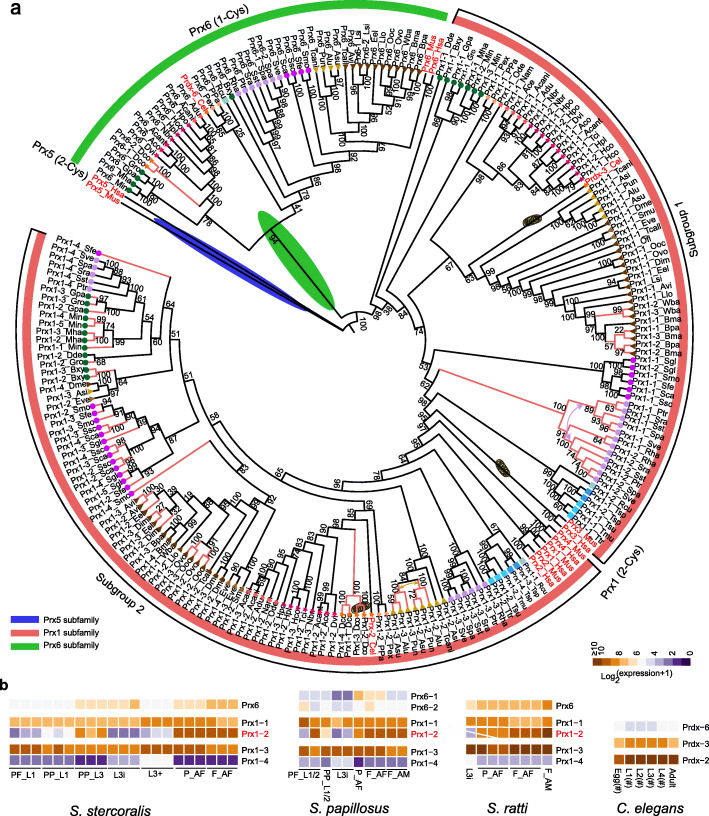
Phylogenetic analyses of PRX in different nematodes and mammals. **a** Unrooted phylogeny of PRX in nematodes and mammals. Shape and color leaf decoration refer to clades and subclades, respectively, as depicted in Fig. 2. **b** Expression of PRX in four nematodes. F_ indicates free-living, P_ is parasitic, AF is adult female, AM is adult male, PP is post parasitic, PF is post free-living, and i is infective. Number sign indicates RPKM

The number of conserved active cysteine residues in mammalian PRX can be classified into three groups: 1-Cys PRX (Prx6), typical 2-Cys PRX (Prx1), and atypical 2-Cys PRX (Prx5) [54]. In phylogenetic analyses, nematode PRX clustered into two groups of 1-Cys (Prx6) and typical 2-Cys (Prx1) with mammalian PRX (Fig. 6a). Nematodes were found to lack an ortholog of mammalian Prdx5. Prx6 was absent in Trichocephalida (Clade I), guinea worm (Clade IIIc), and pinworm (Clade IIIa).

In the Prx1 subfamily, nematode PRX clustered with human and mouse PRDX1-4 (Fig. 6a). The orthologs could be separated into two subgroups based on phylogeny. In subgroup 1, nematode PRX clustered with mammalian PRDX3, including Prdx-3 from *C. elegans* (Fig. 6a). Prdx-3 from *C. elegans* and mammals has a major location in the mitochondrion. We observed that this group of Prx1 in Clade IVb had two copies (Prx1-1 and Prx1-2) and originated in their last common ancestor (Fig. 6a). Gene expression profiles of three *Strongyloides* species showed that Prx1-1 has stable expression, while the expression of Prx1-2 was low in the infective stage but with higher expression in adults (Fig. 6b). One (Prx1-3) of two divergent *Strongyloides* Prx1 subgroup 2 copies was shown to have high expression, while the other (Prx1-4) had low expression (Fig. 6b). Although *D. coronatus* lacked Prx1 subgroup 1, four items from Prx1 subgroup 2 were present, two of which were predicted to be localized in mitochondria (Fig. 6b). It has been shown that cytosolic Prdx-2 (Prx1 subgroup 2) from *C. elegans* is more important for protecting against H_2_O_2_ than Prdx-3 (Prx1 subgroup 1) and is expressed in several tissues, including the intestine [55]. In *C. elegans*, *Prdx-2* had higher expression than *Prdx-3* in developmental stages (Fig. 6b). The transcriptome data of other parasitic nematodes also showed higher expression of Prx1 in subgroup2 than in subgroup1 (Additional file 6: Table S6). This evidence suggests the importance of cytosolic Prx1 in defending against H_2_O_2_.

## Discussion

Nematodes are one of the most abundant groups of animals on Earth and have existed since the Palaeozoic. Nematodes have evolved variable defense system to live in diverse habitats and ecological niches in lifestyles of free-living, facultative parasitic, and obligate parasitic forms. They have also experienced independent evolution to adapt to the environment, animals or plants at least several times [1]. Nematodes make use of antioxidant enzymes to defend against endogenous (metabolic processes) and exogenous (host or environment) ROS [4]. However, the origin and evolution of antioxidant enzymatic system across the phylum Nematoda remain elusive. In this study, the large dataset of nematode genomes and transcriptomes enabled us to deeply investigate how antioxidant enzymes evolve to adapt diverse ecological niches.

### Overview of antioxidant enzyme families in nematodes and mammals

**Fig. 7:**
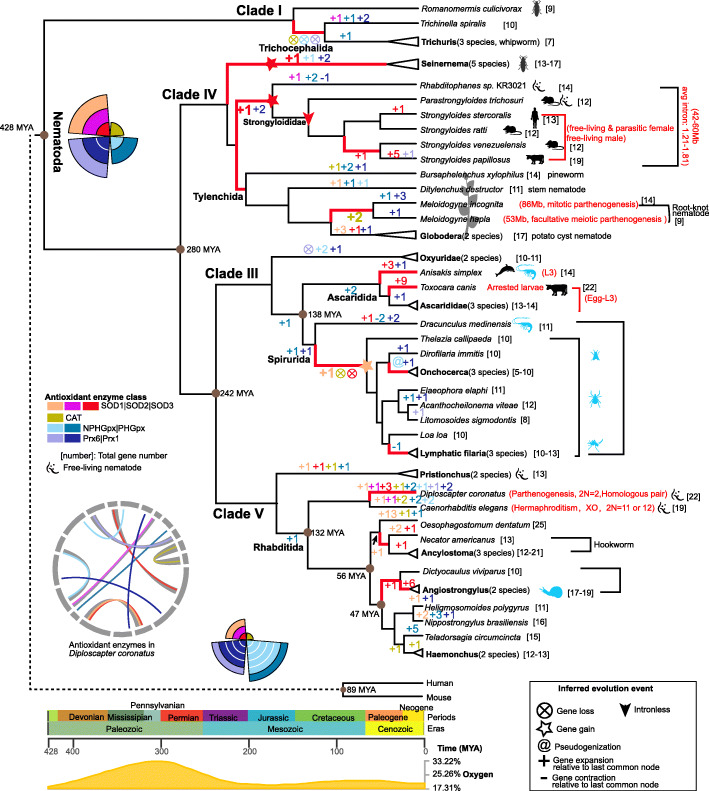
Patterns of antioxidant enzyme evolution in the Nematoda. Antioxidant enzymes evolve mainly through expansion, constriction, gain and loss, and pseudogenization in nematodes. The rose diagram shows the antioxidant enzyme family numbers inferred for the ancestors of nematodes and mammals. The circos plot depicts paired antioxidant enzymes in *D. coronatus*. The numbers in the node represent changes in the gene family relative to the last common node. The color of the number depicts different antioxidant enzymes. The brown dot shows the estimated divergence time retrieved from the TimeTree database and our previous study. Cartoon for species parasitic definitive host or intermediate host was the same to Fig. 2. See Additional file 1: Fig. S17 for detailed antioxidant enzyme numbers for the branches. Antioxidant enzymes in nematodes with red bold branches were detailly discussed

We analyzed 294 SOD, 62 CAT, 206 GPx, and 211 PRX in 59 nematode species, and including several species from per subclade to provide more details for the evolutionary history inference of antioxidant enzymatic system in the phylum Nematoda. We also systematically classified antioxidant enzymes into several families in the phylum Nematoda based on comparative analyses and enzymatic characters. Dynamic changes in the antioxidant enzymes (SOD: SOD1, SOD2, and SOD3; CAT; GPx: PHGPx and NPHGPx; PRX: Prx1 and Prx6) were inferred by considering the phylogenies of the gene families, species, assembly, and annotation issues (change relative to the last common node is shown in Fig. 7**,** and detailed inferred number is shown in Additional file 1: Fig. S17). Nematodes lacked mammalian *Gpx7*, *Gpx8*, and *Prdx5*, and the ancestor of nematodes had a smaller number of NPHGPx (with an inferred one instance in nematodes, and five in mammals) and Prx1 (two in nematodes, and four in mammals, Fig. 7 and Additional file 1: Fig. S17).

The number of antioxidant enzyme in nematodes, unlike the number in mammals, varies in different clades, specifically in Clades III, IV, and V. It has been estimated that the split between Clade III, and Clades IV and V was about 280 mya [22], when oxygen levels increased (oxygen data were retrieved from the TimeTree database [56], Fig. 7). We observed that CAT, Prx6, and NPHGPx were completely lost in the order Trichocephalida (Clade I) and in the families Oxyuridae, Thelaziidae, and Onchocercidae (Clade III), which suggests that these enzymes may not be essential for all of parasitic nematodes, or there may be alternative for them through other H_2_O_2_ scavengers. The pseudogenization of NPHGPx in the genus *Onchocerca* was also found, consistent with previous reports [4]. Contracted gene numbers of PHGPx or Prx1 were also observed in the species *Rhabditophanes* and *D. medinensis*, as well as in lymphatic filaria (Fig. 7). Potential novel extracellular SOD isoforms (SOD1-like and SOD3-like) were revealed using comprehensive analyses of a large array of diverse nematodes. Extensive lineage-specific expansion (5–13) of SOD1 and/or SOD3 were observed in the genus *Angiostrongylus*, species *S. paillosus*, *T. canis*, and *O. dentatum* (Fig. 7). Available transcriptomes showed that lineage-specific expanded genes exhibited stage-specific high expression (Fig. 4a), suggesting that at least part of these may be preserved by natural selection for gene dosage (SOD3 in *Angiostrongylus*, *T. canis*, and *S. paillosus*).

Investigation of free-living nematodes of Rhabditoidea (*C. elegans* and *D. coronatus*) showed that most of antioxidant enzyme families have multiple copies (Fig. 7). Despite the close relationship of these two species, their genomes and biology, including karyotype and reproduction, are different [57]. The duplication mechanism for antioxidant enzymes of these two nematodes differs, in that *C. elegans* features segment duplication, while *D. coronatus* shows a pattern similar to “whole-genome duplication” [58] (circos plot in Fig. 7).

### Crucial species nodes provide novel insights into the origin and evolution of extracellular SOD

The origin of extracellular SOD is not clearly understood. The order Spirurida (Clade IIIc) includes the superfamilies, Chitwoodchabaudiidae (including Dracunculoidea) and Seuratidae (including Thelaziidae and Onchocercidae) [59]. The life cycles of Thelzioidea and Onchocercidae are similar to each other, in that both require a sucking arthropod as an intermediate host, and both may have arisen during the Eocene, while the life cycles found in Dracunculoidea require copepods as the intermediate host and may have arisen as early as the Triassic or the Jurassic [59]. Thus, *D. medinensis*, *T. callipaeda*, and various filaria provide an appropriate timescale to investigate this evolution. We observed that extracellular SOD isoform (SOD1-like) in *T. callipaeda* and 11 filarial worms clustered together with their intracellular isoform (SOD1), while the extracellular isoform (SOD3) in *D. medinensis* clustered together with that from other nematodes (Figs. 3a and 4a). SOD1 and SOD1-like were neighboring and conserved in the gene order in Filarioidea. Further, a different evolutionary rate (branch length, amino acid identity, and genomic identity) was observed in the comparison between SOD1 and SOD1-like and between SOD1-like from lymphatic filaria (e.g., *B. malayi*) and other filarial nematodes (e.g., *O. volvulus*). The identity between SOD1 and SOD1-like in filaria was higher than that for other nematodes (such as SOD1 and SOD3 in *C. elegans*) at the amino acid and genome levels. These evidences strongly support the contention that SOD1-like was duplicated from SOD1 isoform and underwent rapid evolution in filaria. Extracellular SOD in filaria also provides an excellent instance to describe its origin and evolutionary trajectory. It has been shown that extracellular SOD diverged from the intracellular isoform at an early stage of evolution, which occurred before the appearance of plants, fungi, and metazoans [60]. Thus, we speculate that the divergent extracellular SOD (SOD3) may have been presented in their last common ancestor and underwent lost, and then SOD1 recently underwent tandem duplication and diverged into extracellular SOD.

The nematodes in Clade IVa include entomopathogenic nematodes *Steinernema*. Nematodes in Clade IVb include taxa with diverse lifestyles, including free-living (*Rhabditophane*), facultative parasitic (*Parastrongyloides*), and obligate parasitic (*Strongyloides*) forms. We observed a putative novel extracellular SOD isoform (SOD3-like) that may independently appear in the Clades IVa and IVb (Fig. 7). We showed that SOD3-like lacked metal-binding sites and may not bind Cu and Zn (Fig. 4d, Additional file 1: Figs. S11 and S12). Cu/Zn SOD requires Cu for catalysis and Zn to enhance catalytic efficiency and stabilize the protein and is widespread from the periplasm of bacteria to virtually every organelle in the human cell [61]. In addition, a large amount of extracellular Cu-only SODs have been identified in fungi [61], indicating the possibility that metal-independent extracellular SOD may also exist. Transcriptome data from three *Strongyloides* species and two *Steinernema* species showed a relatively higher expression of SOD3-like in larvae than in adults in *Stronglyloides*, and a relatively low expression one in *Steinernema* (Fig. 4e and Additional file 6: Table S6), which implies a potential functional SOD3-like in *Strongyloides*. Phylogenetic analyses (Fig. 4a) also showed divergent and rapid evolution of SOD3-like in *Steinernema*. Further functional experiments to establish the activity and stability of metal-independent SOD3-like are required. In addition to SOD3-like in Clade IVb, extracellular Cu/Zn SOD (SOD3) is also present. A close relationship and similar gene structure were found between SOD3-like in free-living *Rhabditophanes* (three CDSs) and parasitic Strongyloididae (three CDSs), while the phylogenetic relationship and gene structure of SOD3 were divergent (three CDSs for *Rhabditophanes*, but one CDS for Strongyloididae, Additional file 1: Figs. S9 and S10). SOD3 in Strongyloididae underwent extensive intron loss, with extremely high expression in the parasitic stage. This result suggests that SOD3-like does not originate from SOD3 and experienced a slow evolutionary rate in Clade IVb. The origin of putative SOD3-like is still not understood, and more data are necessary.

### Evolution of antioxidant enzymes associated with adaptive evolution across the phylum Nematoda

Adult *Trichuris* (whipworm) parasites in Clade I possess a specialized morphology (whip-like) that the slender anterior part (stichosome) of the body is burrowed within the intestinal epithelium of the host, while the bulbous posterior end of the body lies free in the intestinal lumen of the host, and the anterior region of the whipworm is closely contacted with host intestinal cells and immune system [16]. Transcriptome [16, 17] of adult anterior and posterior body in two *Trichuris* species showed consistently significant upregulation (GFOLD > 1) of SOD3 in anterior (mixed sex) than posterior (female and male) region, with log2-transformed fold change of 2.77–2.88 and 2.85–3.22 in *T. suis* (without biological replicate) and *T. muris* (three biological replicates), respectively (Additional file 6: Table S6). This result supports the importance of SOD3 in parasite-host interaction.

In Clade IV, the striking biology of the genus *Strongyloides*, its female-only parasitic stage and the free-living female and male, provide insights into the genetic basis and evolution of parasitism. Evolutionary analyses of antioxidant enzymes showed that only SOD3 exhibited divergence between free-living *Rhabditophanes* and parasitic Strongyloididae (Fig. 4a). In details, low amino acid identity, lack synteny in gene order that contains SOD3, complete intron loss in the ancestor of parasitic Strongyloididae after diverged from the last common ancestor of *Rhabditophanes*-*Parastrongyloides*-*Strongyloides* clade support sequence divergence of SOD3 between free-living *Rhabditophanes* and parasitic Strongyloididae. Transcriptome of three *Strongyloides* species showed a specific high expression of some copies of SOD3 in the parasitic stage (Fig. 4a and Additional file 6: Table S6), and higher expression of SOD3 in protein level in *S. ratti* parasitic stage compared to free-living ones, and presence in ES of *S. ratti* [23], providing a strong support that SOD3 may also be related to parasitism in *Strongyloides*. Other antioxidant enzymes in species from Clade IVb showed consistency with the speciation and expression in *Strongyloides* had little difference between the free-living and parasitic stages (Additional file 6: Table S6). Taken together, we propose that SOD3 might be the major antioxidant enzyme in Strongyloididae related with its survival in the host.

The nematodes in Clade Vd include Dictyocaulidae (bovine lungworm) and Angiostrongylidae (rat lungworm and *A. costaricensis*). The life cycle of the genus *Angiostrongylus* is complex, involving a definitive host and intermediate host or paratenic host [62], while the life cycle of *D. viviparus* is simple. Our analyses showed that two copies of SOD3 existed in their last common ancestor, but the genus *Angiostrongylus* had lineage-specific expansion of SOD3 (at least 6 genes). Further, our previous study [63] and transcriptome data from *A. cantonensis* and *D. viviparous* showed that ancestor SOD3 had a higher expression in the mammalian stage (Fig. 4a). Localized expression of extracellular SOD3 was observed at the cuticle and around the intestine in *A. cantonensis* [63], or hypodermis localization in *B. malayi* and *O. volvulus* [37, 64]. Extracellular Cu/Zn SOD activity was detected in the ES of *A. cantonensis* [65]. It can infer that extracellular Cu/Zn SOD might be easily secreted into the extracellular matrix or ES for defense against host ROS. Lineage-specific expansion was found in *Angiostrongylus* and was absent in its close taxa, and relatively high expression was found in the snail-borne stage, all of which support our previous hypothesis that lineage-specific expansion of SOD3 may promote its survival in its snail host [22].

In addition, comparison of antioxidant enzyme expression in adult female from parasitic and free-living stages of three *Strongyloides* species, or in adult anterior and posterior body from two *Trichuris* species, showed most antioxidant enzymes (including SOD1, SOD2, CAT, PRX) were not significantly differential expression, except for SOD3 (Additional file 6: Table S6). It is known that H_2_O_2_ could be across membrane by the diffusion and aquaporin channel to perform functions, while O_2_^−^ is limited [66, 67]. Thus, we proposed that the functions of some antioxidant enzymes, including SOD1, SOD2, CAT, PRX, might maintain the low physiological concentrations of ROS for a series of physiological activities of nematodes.

## Conclusions

In summary, we provide the first systematic annotation and classification of antioxidant enzymes for nematodes. Comparative analyses of antioxidant enzymes in the phylum Nematoda provided novel insights into the origin of extracellular SOD and its evolutionary trajectory, and it revealed a novel metal-independent putative extracellular SOD3-like. Further, the close relationship of nematodes with diverse life cycles or lifestyle also provide some evidences for the lineage-specific expansion of extracellular SOD3 related to the complex life cycles of nematodes, and extracellular SOD3 may be beneficial for parasitism. Alternative enzymes neutralizing H_2_O_2_ (CAT, GPx, and PRX) showed that some had been lost and that there are minor variations in their number and expression across parasitic nematodes. Our study provides a deep understanding of the evolution of antioxidant enzymes in nematodes, which could provide targets for anthelmintic control.

## Methods

### Genome-wide identification of antioxidant enzymes

In all, 103 nematode assemblies (86 species), not including *A. cantonensis* [22] and *N. brasiliensis* [34], were retrieved from Wormbase WBPS10 [68]. We filtered parasitic species with low assembly metrics (Scaffold N50 and Contig N50 < 1 kb), and we retained one high-quality assembly for species with multi-assemblies. We chose *C. elegans* and *T. spiralis* as representatives for the genus *Caenorhabditis* and *Trichinella*, respectively (59 species, Additional file 2: Table S3). To avoid the introduction of differences by different annotation methods from original genome annotations, we employed a uniform pipeline to identity antioxidant enzymes across nematode genomes, with the exception of well-studied *C. elegans*. This pipeline was similar to the olfactory receptor gene annotation done for the seahorse genome [69]. Simply as following: first, we downloaded known helminth antioxidant enzymes deposited in the Swiss-Prot database as queries for baiting homologies in the nematode genomes. Then, these queries were aligned for each genome using BLAST [70] (v2.2.26) with parameters “-p tblastn -F F -e 1e-5.” Solar [71] software (v0.9.6) was used to join high-score blocks between each pair of protein mapping results, and alignment rates of less than 0.5 were discarded. Subsequently, protein sequences were mapped to the genome using GeneWise [72] (wise package, v2.2.3) and extended 500 bp upstream and downstream to define integrated gene models. The domain was predicted from a search of the Pfam database (v28.0) using the program HMMER [73] (v3.1b2) with an *e* value less than 0.001. Domains encoded in different antioxidant enzymes from *C. elegans* were used to further discard the fragmentary genes from other nematodes. We also used HMMER to detect potential antioxidant enzymes in the original genome annotation. Then, we manually examined sequences of these antioxidant enzymes in original gene annotation and reannotated gene annotation by checking information, such as gene length, domain annotation, and blast hit information. All antioxidant enzyme information discussed in this study is listed in Additional file 3: Table S4, and reannotated sequences of antioxidant enzymes in 58 nematodes are listed in Additional file 4. Signal peptide was inferred by Phobius [74] (v1.01). Multiple sequence alignment was edited with Jalview [75] (v2.11.0). The 3D protein structure for SOD3 (4oja as the template) and SOD3-like (1n19 as the template) of *S. ratti* were predicted using the online SWISS-MODEL (https://swissmodel.expasy.org). The 3D structures of the protein for SOD3 from human and *H. contortus* were retrieved from SWISS-MODEL. Protein structure visualization was performed using PyMOL (http://pymol.org).

### Comparative genomic analysis

Phylogenetic relationship of 59 nematodes was inferred [10, 24, 36]. Divergence times for several species was retrieved from the TimeTree database [56] (http://www.timetree.org/) and our previous estimation [22]. Oxygen content change was retrieved from the TimeTree database. To study the evolution of antioxidant enzymes across species in the phylum Nematoda, we performed phylogenetic analyses for each gene family. Corresponding genes from human and mouse were used as an outgroup, except for SOD3 and PRX. Firstly, we used MUSCLE [76] (v3.8.31) to perform multiple sequence alignment based on protein sequence. Poor alignment was trimmed with TrimAl [77] (v1.2). Then, IQ-TREE [78] (v1.6) was employed to select the best model for Maximum-Likelihood and reconstruct phylogenetic trees. Visualization were conducted using Evolview [79] (https://www.evolgenius.info/evolview) or iTOL [80] (https://itol.embl.de/). For SOD and GPx gene families, due to unclear resolve class, we first clustered them into orthologous groups using OrthoMCL to remove divergent singleton sequences [81] and then reconstructed the phylogeny for subfamilies independently. Nonsynonymous substitutions per nonsynonymous site (*Ka*) and synonymous substitutions per synonymous site (*K*s) of pairwise pairs were calculated by Yn00 from PAML package [82] (v4.9h). Synteny of genes in different species was performed using MCSCANX [83] (https://github.com/tanghaibao/jcvi) with default parameters. We also used reciprocal best hit (RBH) to detect orthologous genes in two genomes. To estimate ortholog copy number in seven root-knot nematode genomes, first, we employed the BUSCO pipeline [84] to detect orthologs of single-copy genes in nematodes (nematode dataset deposited in OrthoDB v10) with the parameter “-m genome --augustus_species caenorhabditis” (Additional file 1: Table S2). Then, the potential ortholog (single-copy, duplicated and fragmented BUSCO groups were all considered) copy number (1:1, 2:1, ≥ 3:1) relative to the diploid *M. hapla* was summarized (Additional file 1: Table S2 and Fig. S14C).

### Species abbreviation and antioxidant enzyme nomenclature

The species abbreviations consisted of an uppercase initial letter from the genus name and two lowercase initial letters from the species name. If repeated species abbreviations occurred, four lowercase initial letters from the species name were extracted. The newly designated gene names were represented by the name of the antioxidant enzymes family with an underline and three or five letters species abbreviation (e.g. Sod2_Acant for Sod2 from *A. cantonensis*). For multiple gene duplicates, each copy was designated by a dash and a number (e.g., Sod3-1_Acant, Sod3-2_Acant, and Sod3-3_Acant). To avoid confusion and conflict, gene names for *C. elegans* and outgroup (human and mouse) were kept.

### Antioxidant enzyme expression profile analyses

To investigate gene expression pattern of these antioxidant enzymes during the nematode development, we downloaded RNA-seq datasets of the developmental stages from 20 nematodes with data available in SRA database (Additional file 5: Table S5). FastQC (v0.11) was used to check the quality, and Trimmomatic [85] (v0.38) was used to filter low-quality reads. We then aligned reads to the reference genome with HISAT2 [86] (v2.1), and alignment summary information is listed in Additional file 5: Table S5. We used featureCount from Subread package [87] (v1.6) to obtain read count and normalized sequencing depth. FPKM or RPKM was used to normalize expression for paired-end or single-end RNA-seq, respectively. Pearson correlation of samples (or biological replicates) in a species is shown in Additional file 5: Table S5. Hierarchical clustering analysis of gene expression profile was conducted by Pheatmap of R package using “Euclidean distance” as clustering distance method. Gene expression specificity [88] was calculated based on normalized log-transformed expression values across all available data sets. Differential expression analysis was only performed on samples with at least two biological replicates or nematodes with at least two species (available RNA-seq data) in a genus and with sufficient data amount (> 10 million reads) and appropriate overall genome alignment (> 70%). To detect differential expressed antioxidant enzymes among nematode transcriptome data, we employed GFOLD [89] (v1.1.4), a tool for ranking differentially expressed genes from RNA-seq data, which is specifically useful when no replicate is available. Genes with |GFOLD| > 1 were defined as significantly differentially expressed (blue sheet in Additional file 6: Table S6).

## Supplementary information

**Additional file 1: Supplementary Figs. S1 to S17**, and **Tables S1-S2**.

**Additional file 2: Table S3.** Assembly and gene annotation information of 59 nematodes.

**Additional file 3: Table S4.** Gene information of antioxidant enzymes in reannotation and original gene annotation in 59 nematodes.

**Additional file 4:.** Protein-coding transcript (cds) and Protein-coding transcript translation (pep) sequences, and GFF (gff) files of antioxidant enzymes in 58 nematodes.

**Additional file 5: Table S5.** Transcriptome data information (SRA information and genome alignment summary) for 20 nematodes.

**Additional file 6: Table S6.** Expression of antioxidant enzymes in 20 nematode developmental stages and pairwise comparison of antioxidant enzymes in 11 nematodes (blue sheets) detected by GFOLD.

## Data Availability

The datasets analyzed during the current study are available in the public databases (Wormbase and NCBI). All relevant accessions of genomes and transcriptomes are listed in Additional file 2: Table S3 and Additional file 5: Table S5.

## Abbreviations

ROS: Reactive oxygen species
SOD: Superoxide dismutase
CAT: Catalase
GPx: Glutathione peroxidase
PRX: Peroxiredoxin
PHGPx: Phospholipid hydroperoxide glutathione peroxidase
ES: Excretory-secretory
FPKM: Fragments Per Kilobase of transcript per Million mapped reads
RPKM: Reads per kilobase of gene per million mapped reads
