# Supplementary material for: Speciation and adaptive evolution reshape antioxidant enzymatic system diversity across the phylum Nematoda
Source: BMC Biol. 2020 Nov 26;18:181. doi: 10.1186/s12915-020-00896-z (PMC7694339; doi:10.1186/s12915-020-00896-z)
Supplement: Supplementary file 1 — Additional file 1: Supplementary Figs. S1 to S17, and Tables S1-S2. [file 12915_2020_896_MOESM1_ESM.docx]

**Supplementary Figures and Table S1**

**
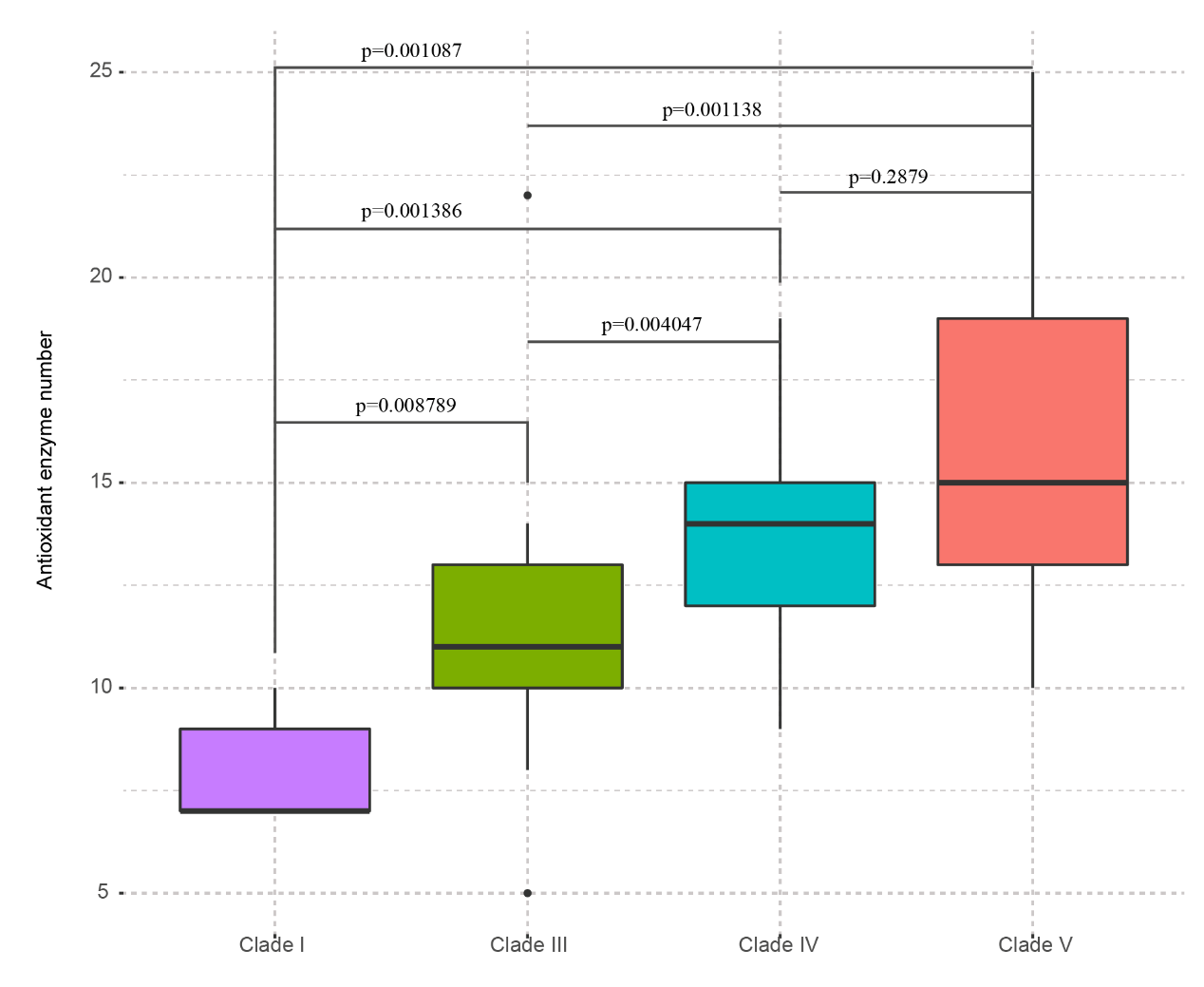
**

**Figure S1. Antioxidant enzyme number in Clade I was significantly less than other clades (p<0.05).** Wilcoxon rank-sum test was performed using the function of “wilcox.test” from R program. Outlier values was shown in black points.


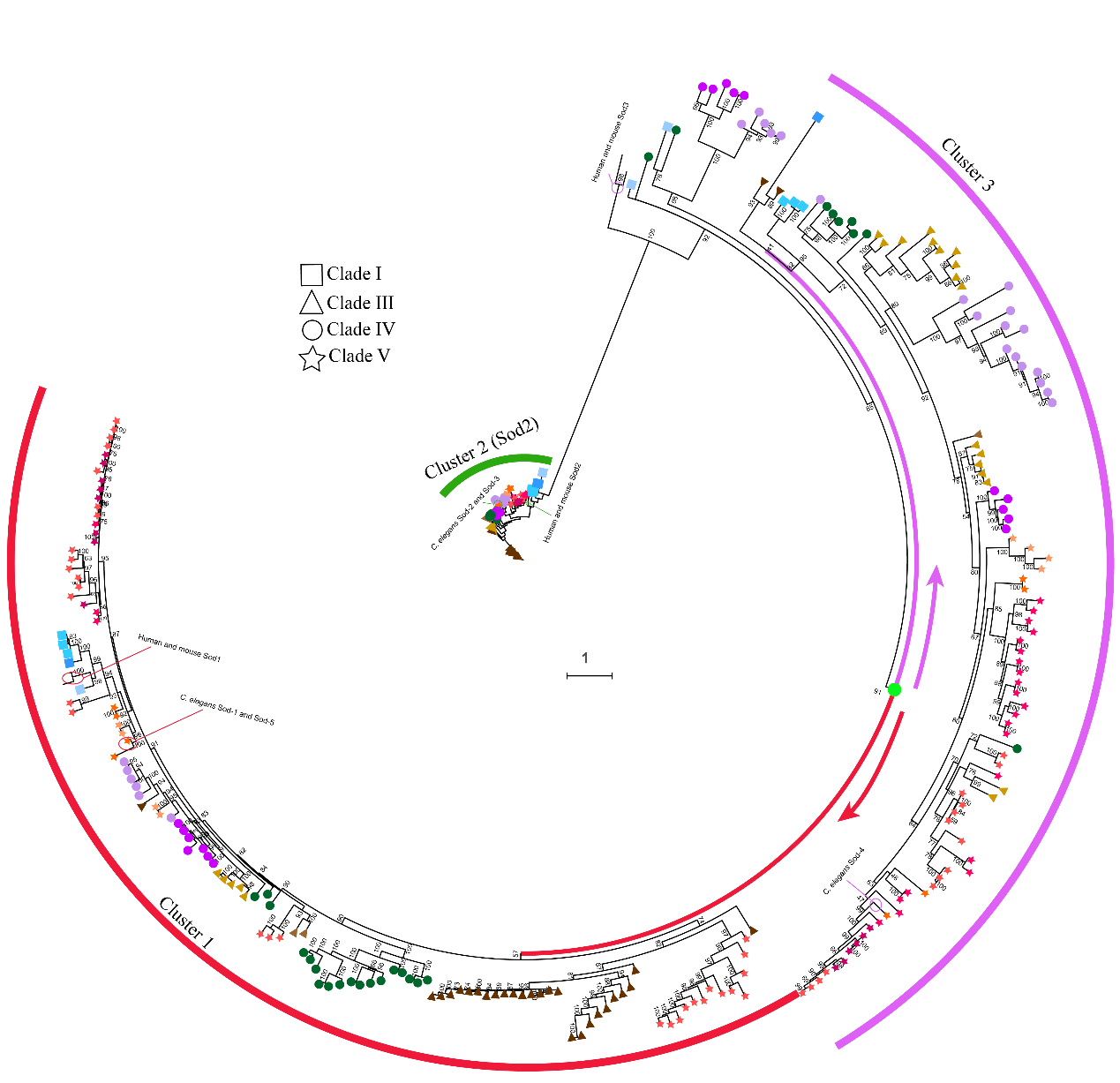


**Figure S2. Phylogeny of SOD gene family based on maximum likelihood** **algorithm**. Three hundred *Sod* genes from nematodes and mammals were divided into three major clusters (Cluster 1, 2 and 3). SOD1 from *C. elegans* (*Sod-1* and *Sod-5*) and SOD1 from human and mouse were included in Cluster 1. SOD3 from *C. elegans* (*Sod-4*) were included in Cluster 3, but with different phylogenetic relationship in Cluster 1. Shape and color leaf decoration depicts clade and subclade information in Fig. 2 in the main text. The scale bar represents the number of amino acid substitutions per site. Bootstraps in cluster 2 were not shown because of too intensive to visible.


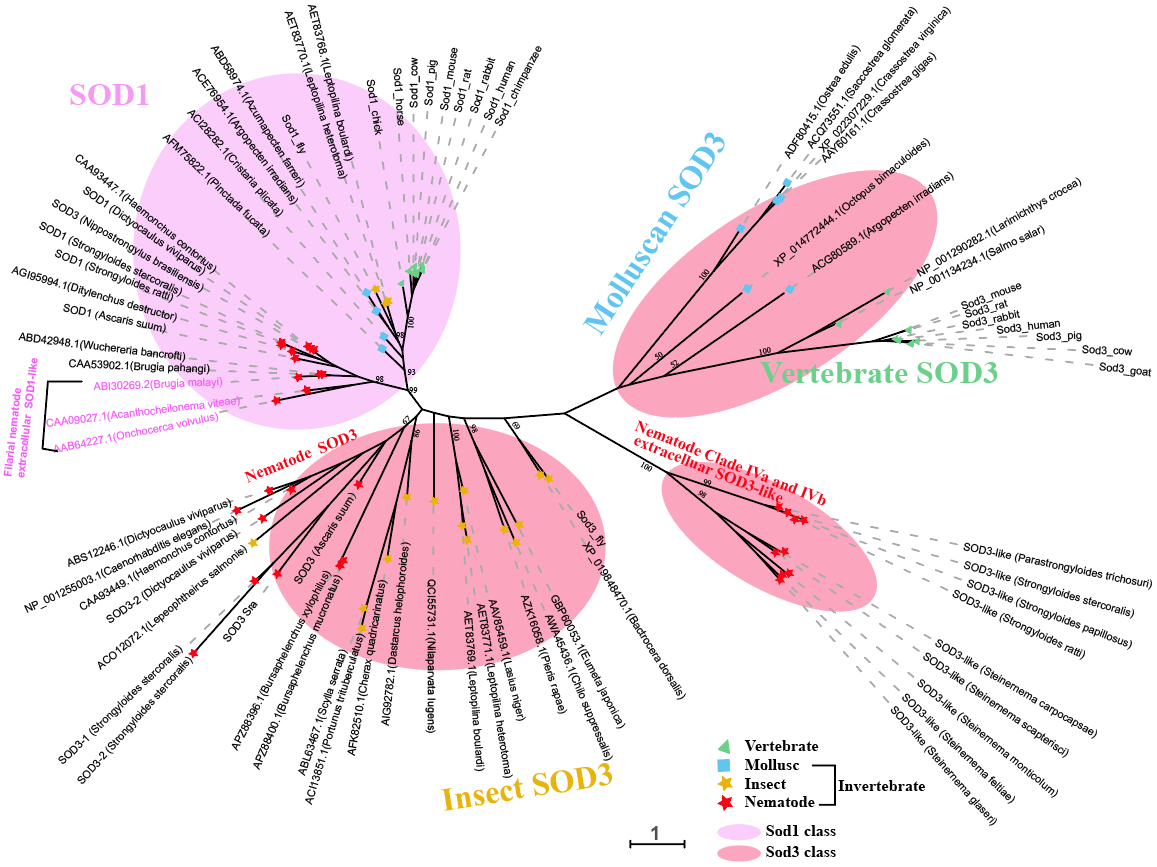


**Figure S3**. **Unrooted phylogeny of Cu/Zn-SOD in vertebrates and invertebrate animals**. Cu/Zn-SOD in vertebrates and invertebrates were collected from public databases (NR and Swiss-Prot). The scale bar represents the number of amino acid substitutions per site.


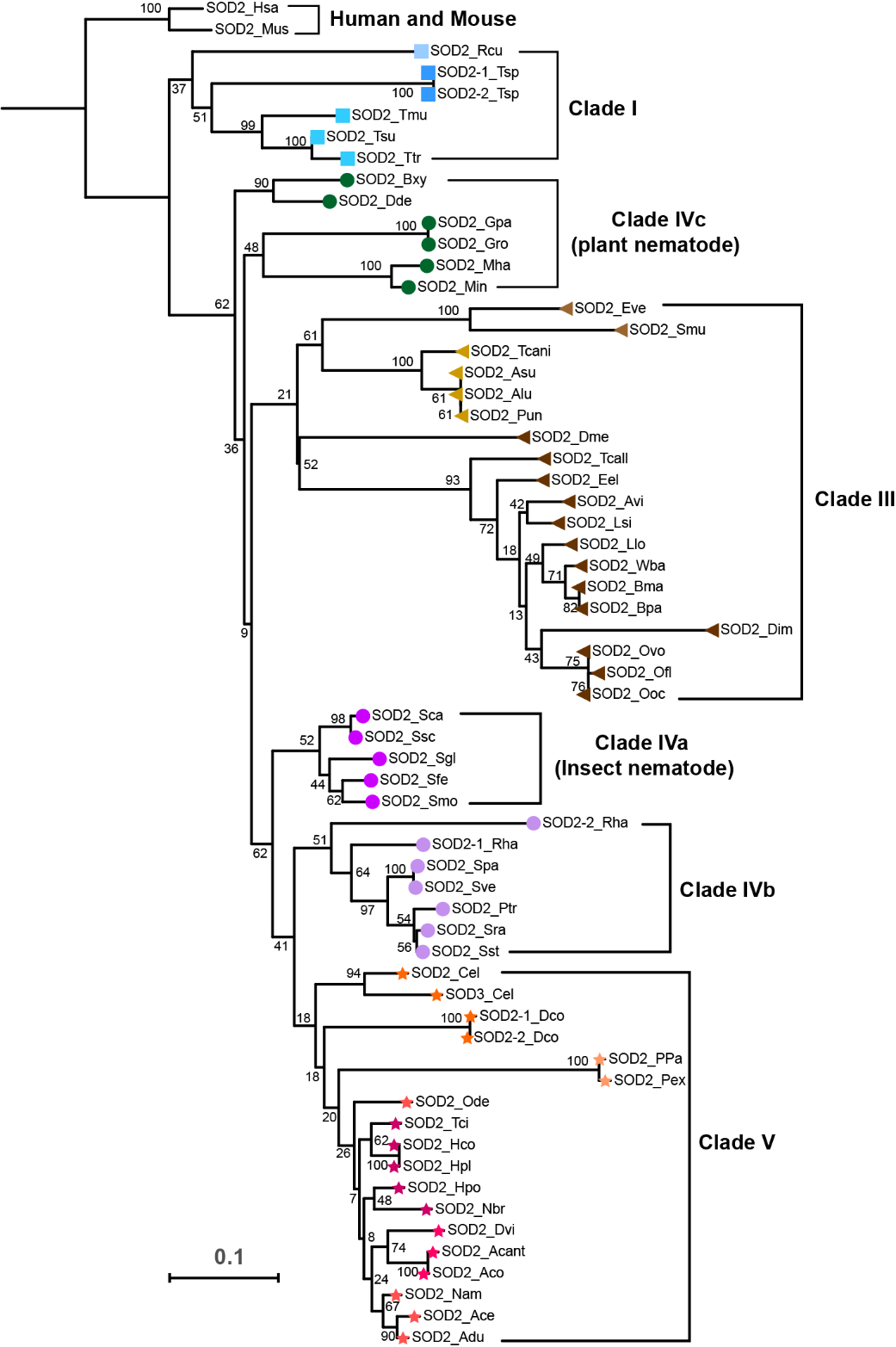


**Figure S4. Rooted phylogeny of 63 SOD2 from nematodes, human, and mouse based on neighbor-joining algorithm**. Three or five abbreviation for species, full name was shown in **Fig. 2** in the main text. The numbers at internal branches showed bootstrap support values. The scale bar represents the number of amino acid substitutions per site. SOD2 from mammal was used as an outgroup.


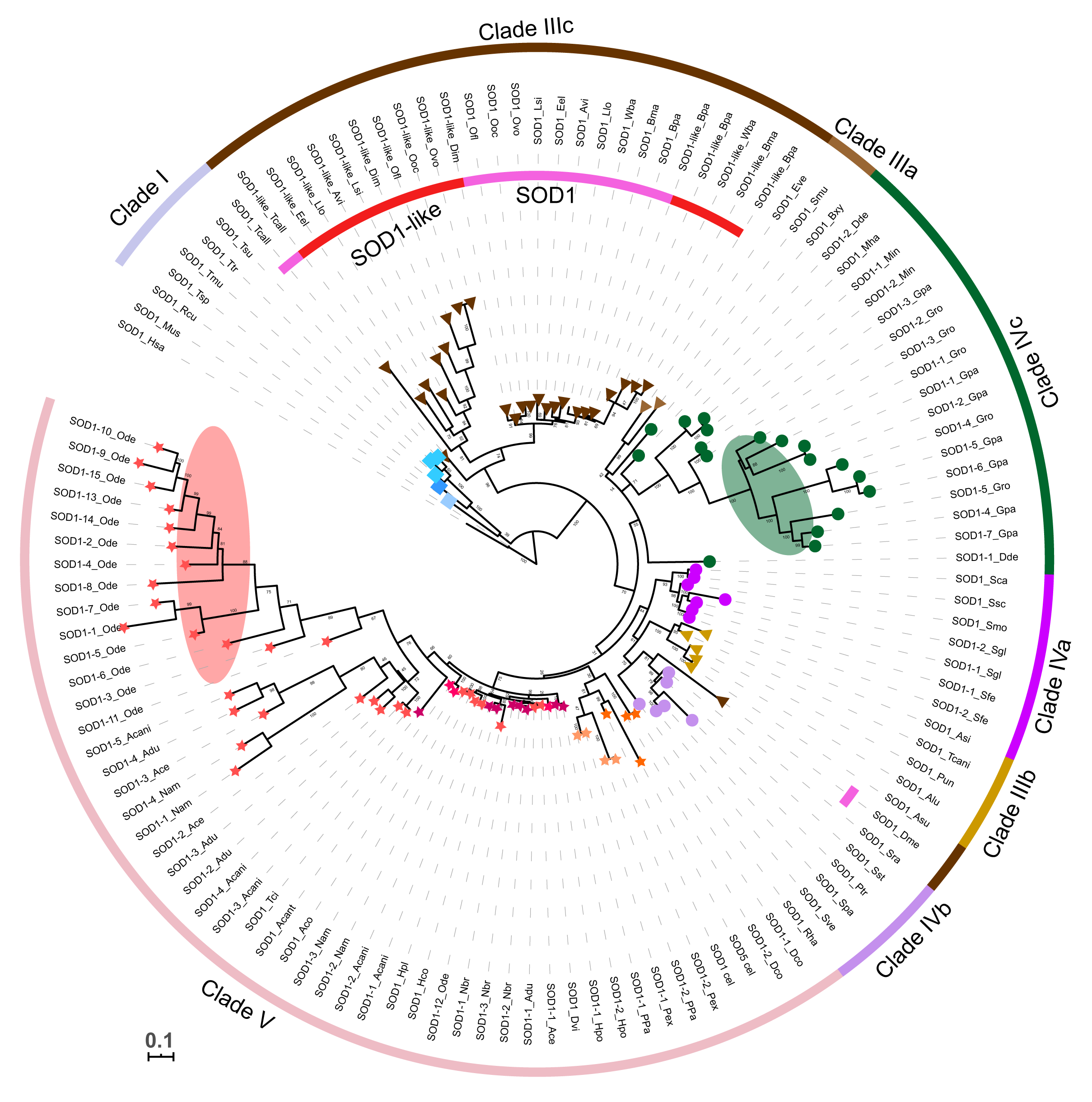


**Figure S5. Rooted phylogeny of SOD1 group from nematodes, human, and mouse based on maximum likelihood algorithm**. Three or 5 abbreviation for species, full name was shown in **Fig. 2** in the main text. The numbers at internal branches show bootstrap support values. The scale bar represents the number of amino acid substitutions per site. Ellipse showed species or lineage-expansion. Red and pink strip in the inner showed SOD1-like or SOD1 in the Clade IIIc. Outer strips showed clade information.


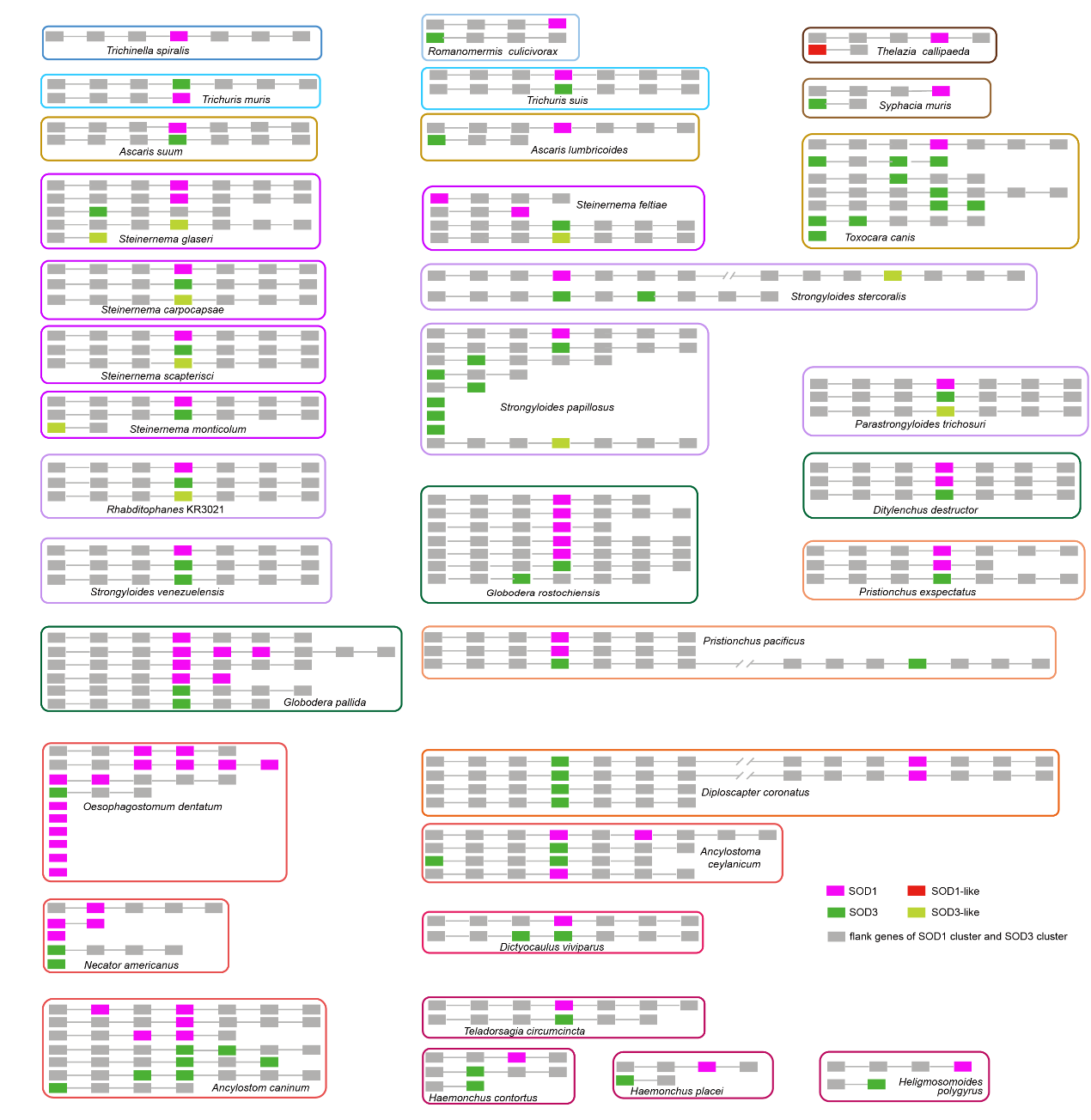


**Figure S6. Gene locus from cluster 1 and cluster 3 of SOD gene family in non- filarial nematodes**. Three up- and down- neighbor genes were shown. Nematodes with too fragment were not shown. This result showed that SOD1 (pink) and SOD3 (green) in non-filarial nematodes were not adjacent.


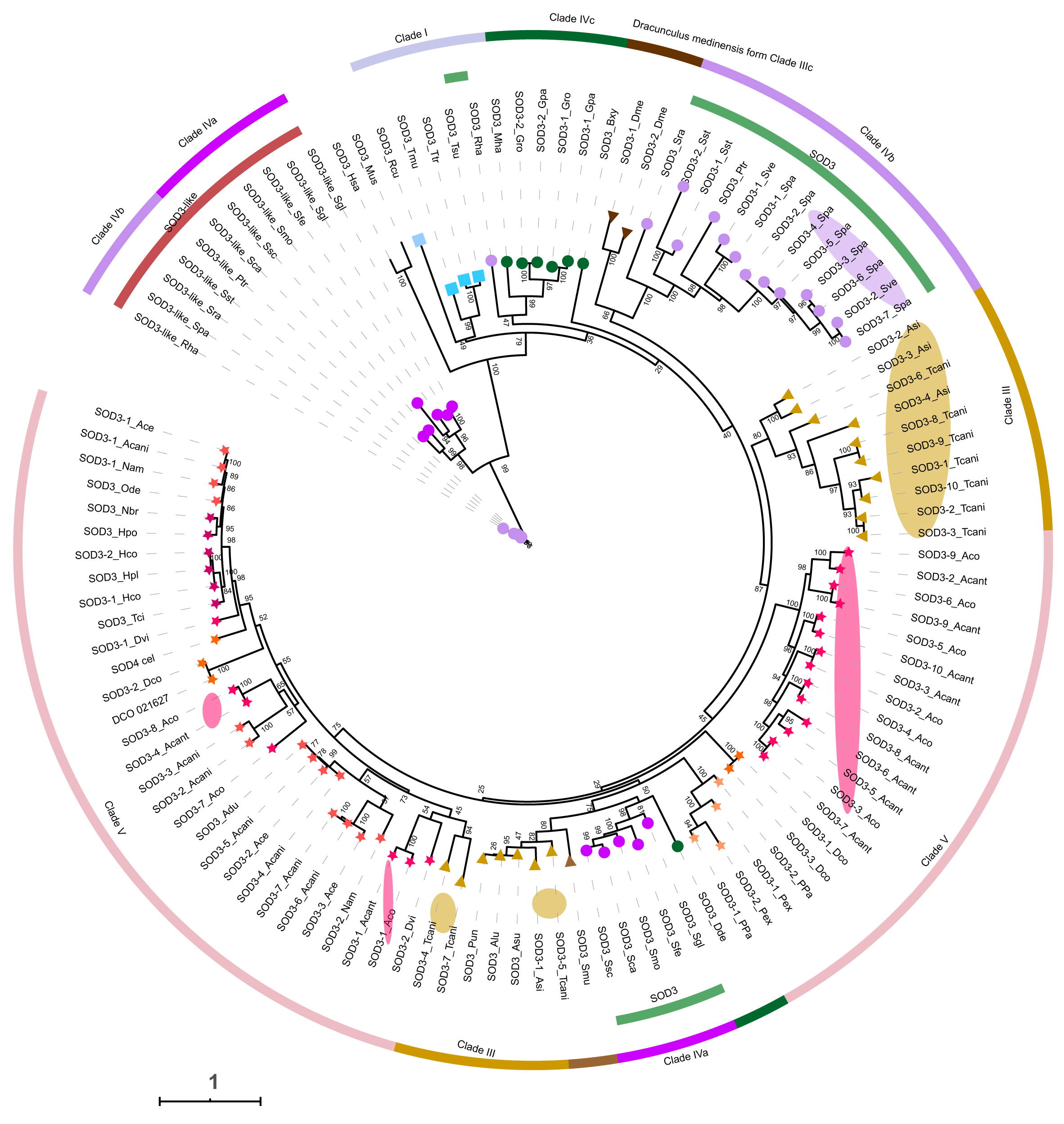


**Figure S7. Phylogenetic analysis of SOD3 group from nematodes, human, and mice**. 3 or 5 abbreviation for species, full name was shown in **Fig. 2** in the main text. The numbers at internal branches show bootstrap support values. The scale bar represents the number of amino acid substitutions per site.


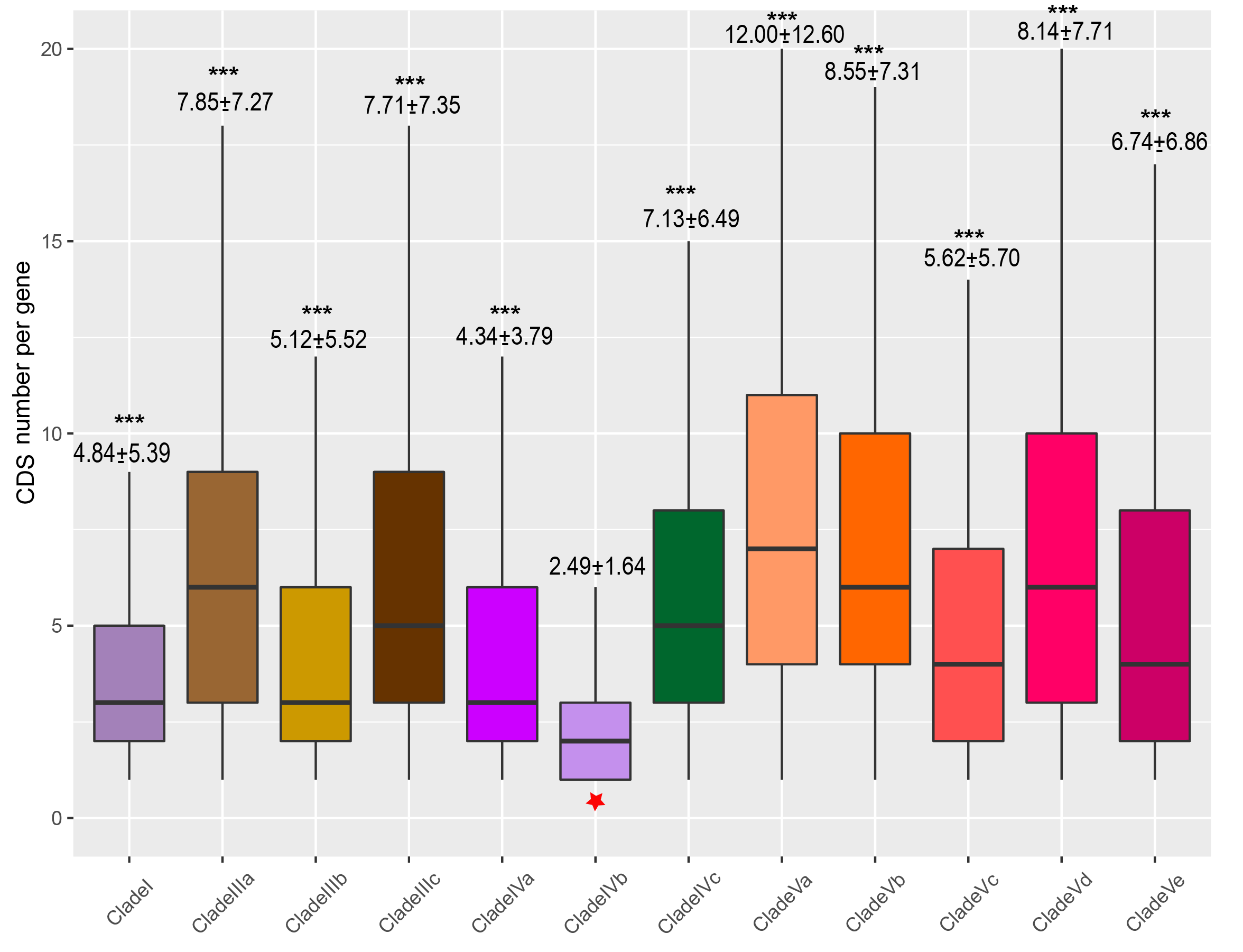


**Figure S8. The distribution of CDS number per gene (whole gene set) in nematodes from Clade I, Clade IIIa, IIIb, IIIc, IVa, IVb, IVc, Va, Vb, Vc, Vd, Ve**. Wilcox test was performed by compared other groups with Clade IVb. *** indicated p<0.001. text above boxplot showed mean and standard deviation.


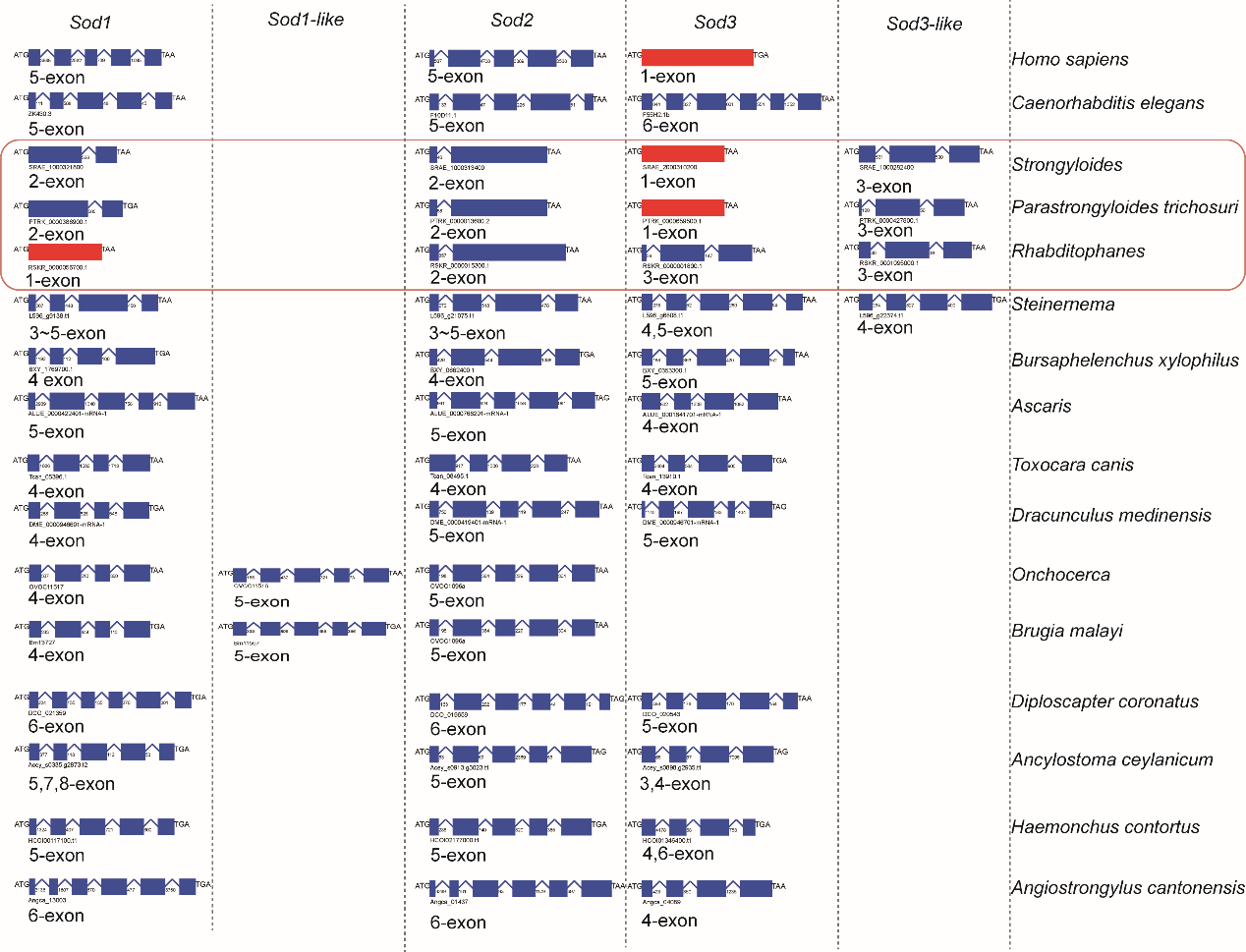


**Figure S9. Gene structure (ORF) of SOD families in human and nematodes**. Genes with complete ORF are shown. If several genes encoded in a species, only one is shown. Red rectangle indicates single CDS and blue rectangle indicate multiple CDSs.


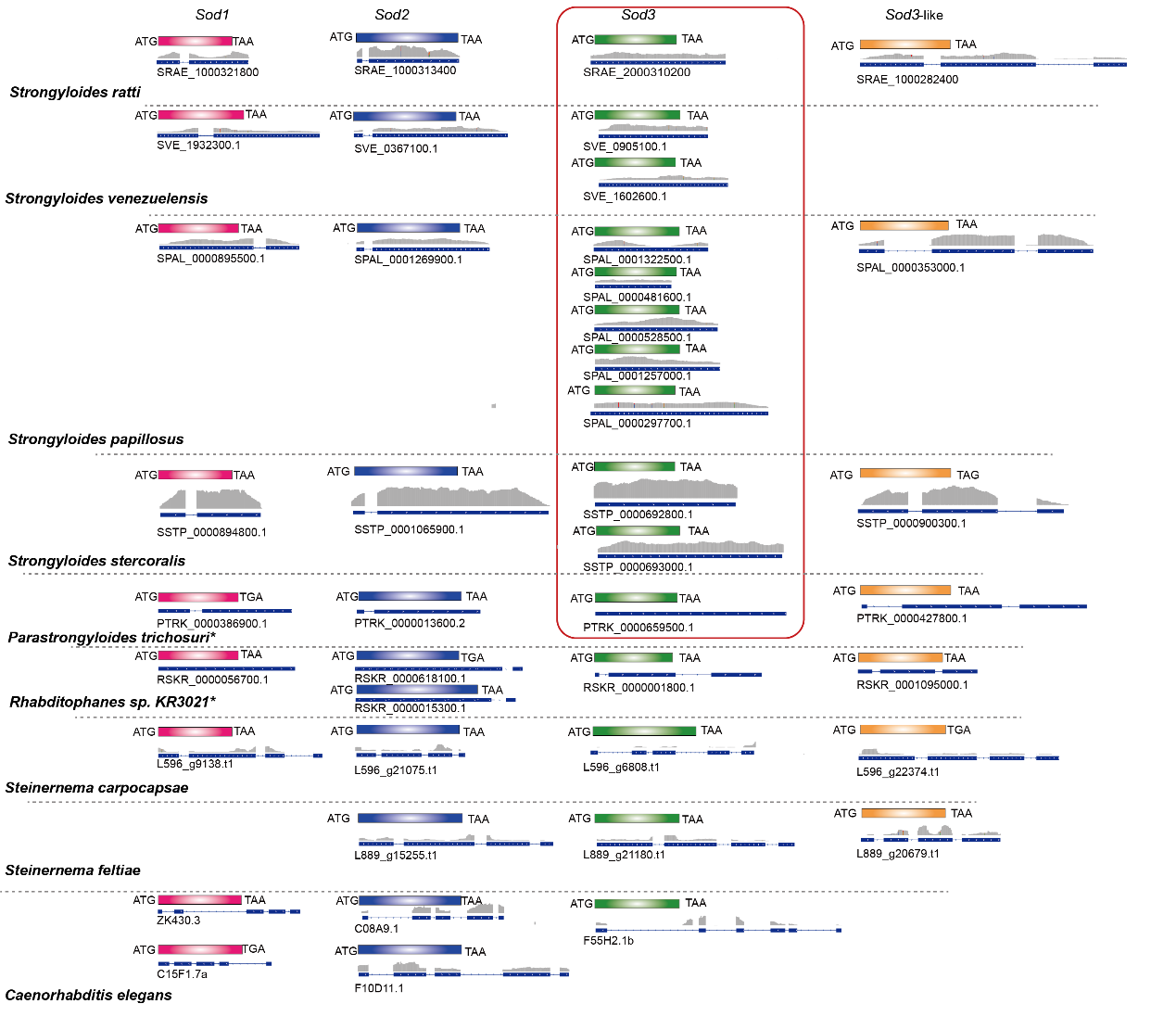


**Figure S10. Gene structure (ORF) of SOD families in *Strongyloides*, *Steinernema* and *C. elegans* with transcriptomic coverage support.** Blue rectangle indicates CDS, blue line indicates intron. Colorful rectangle indicates cds of different SOD gene family member. Star indicates species lacks available RNA-seq data.


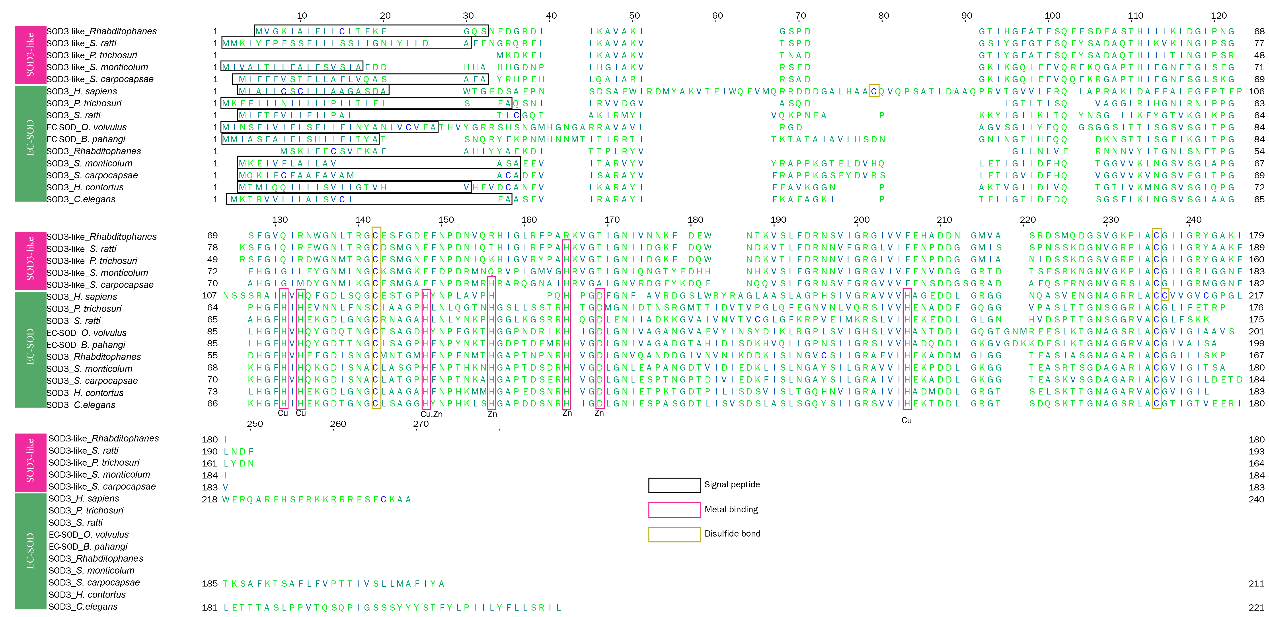


**Figure S11. Multiple sequence alignment of EC-SOD and SOD3-like in nematodes and human. Black box showed signal peptide**. Red box showed metal binding site. Yellow box showed disulfide bond.


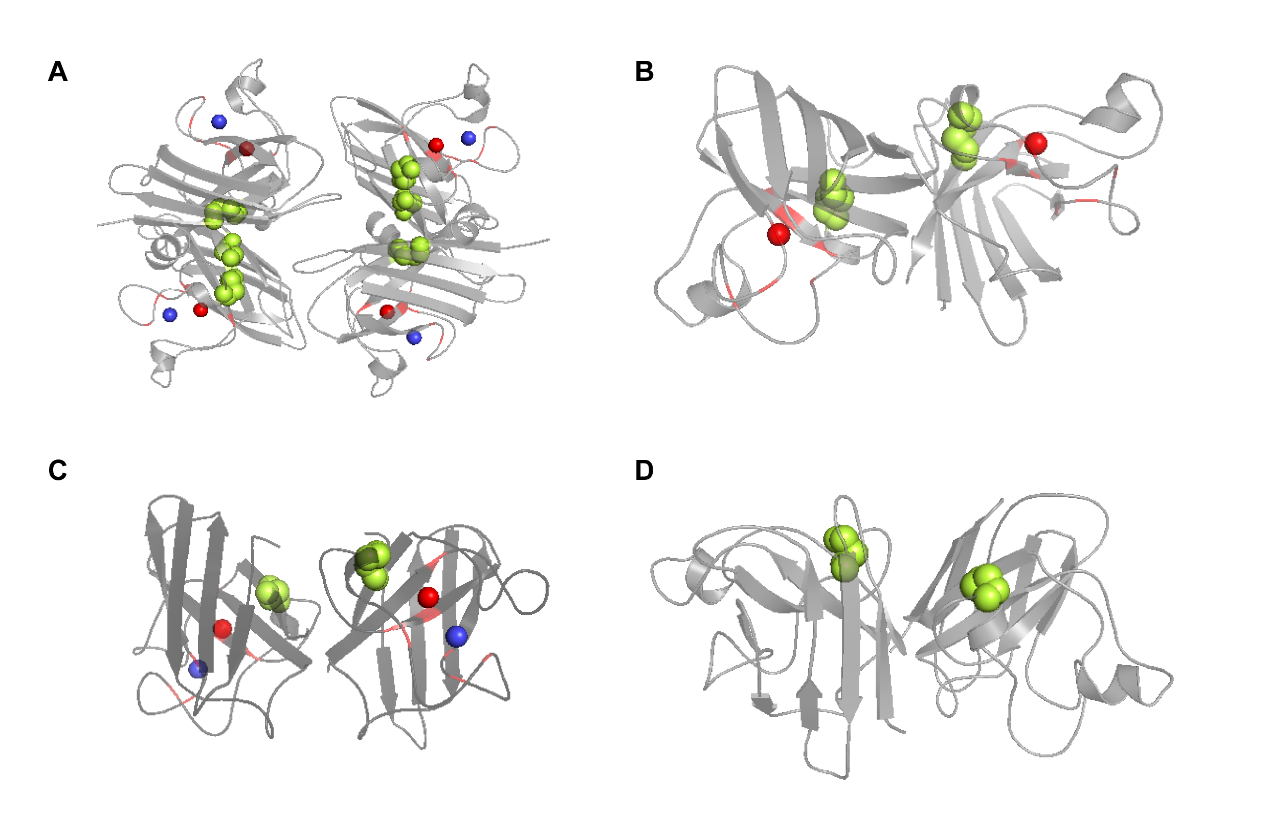


**Figure S12. Protein structure prediction of SOD3 and SOD3-like**. **A**, 3D structure of human SOD3 (2jlp). **B**, 3D structure of *Haemonchus contortus* SOD3 (1sos.1.A as template). **C**, predicted 3D structure of *Strongyloides ratti* SOD3. **D**, predicted 3D structure of *Strongyloides ratti* SOD3-like. **A** and **B** were retrieved from SWISS-MODEL. **C** and **D** were predicted using online SWISS-MODEL. Metal binding sites were highlighted in red. Red and blue ball indicated copper and zinc respectively. Green ball showed disulfide bond.


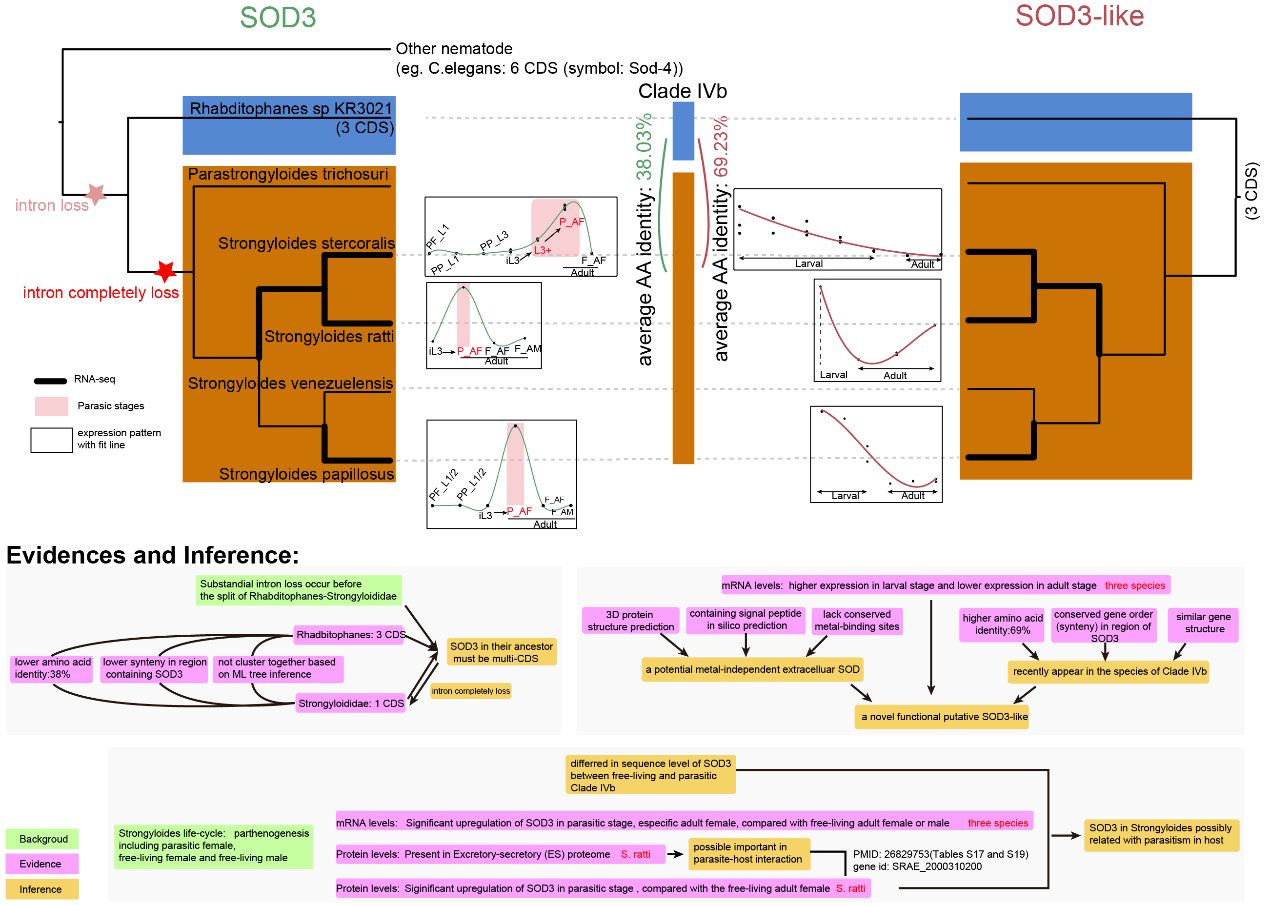


**Figure S13. Illustrated evidences for the discussion that SOD3 and SOD3-like in *Strongyloides*.**


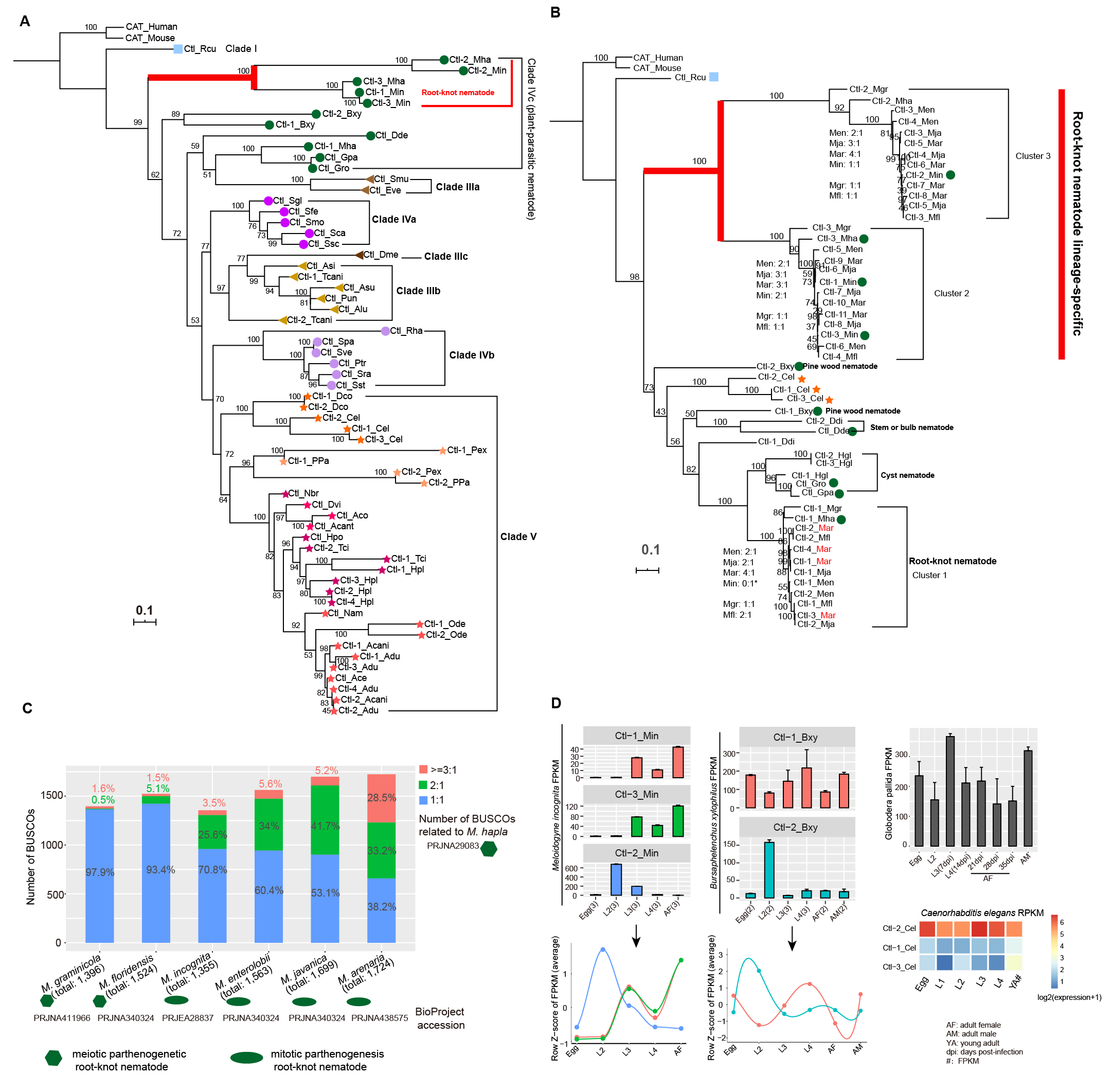


**Figure S14. Phylogenetic analyses of CAT in different nematodes.** **A**. Phylogenetic analysis of CAT in different nematodes. 3 or 5 abbreviation for species name, full name was shown in **Fig. 2** in the main text. The numbers at internal branches show bootstrap support values. The scale bar represents the number of amino acid substitutions per site. **B**. Phylogenetic analysis of CAT in plant-parasitic nematodes and C. elegans. **C**. Ratio (1:1, 2:1, and ≥ 3:1) of BUSCO groups shared between a *Meloidogyne* species and the diploid *M. hapla* (act as the baseline) based on nematode dataset deposited in OrthoDB v10 using BUSCO pipeline. Numbers in parentheses were the share number of BUSCO groups between a root-knot nematode and the diploid *M. hapla*. **D**. Expression profile of CAT across developmental or sexual stages in four nematodes.


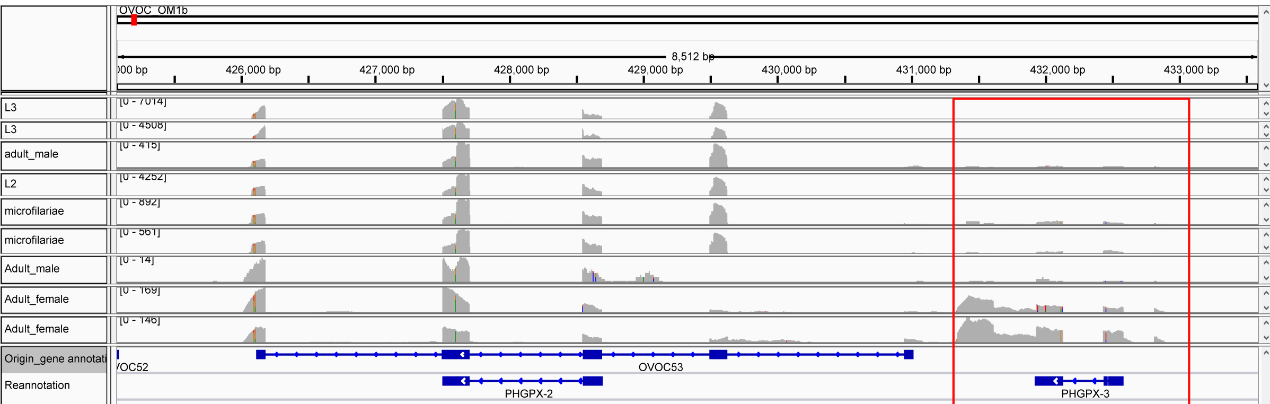


**Figure S15. Transcriptional evidence of presence of PHGPx-3 in the genome of *Onchocerca volvulus* according to RNA-seq data.**

**
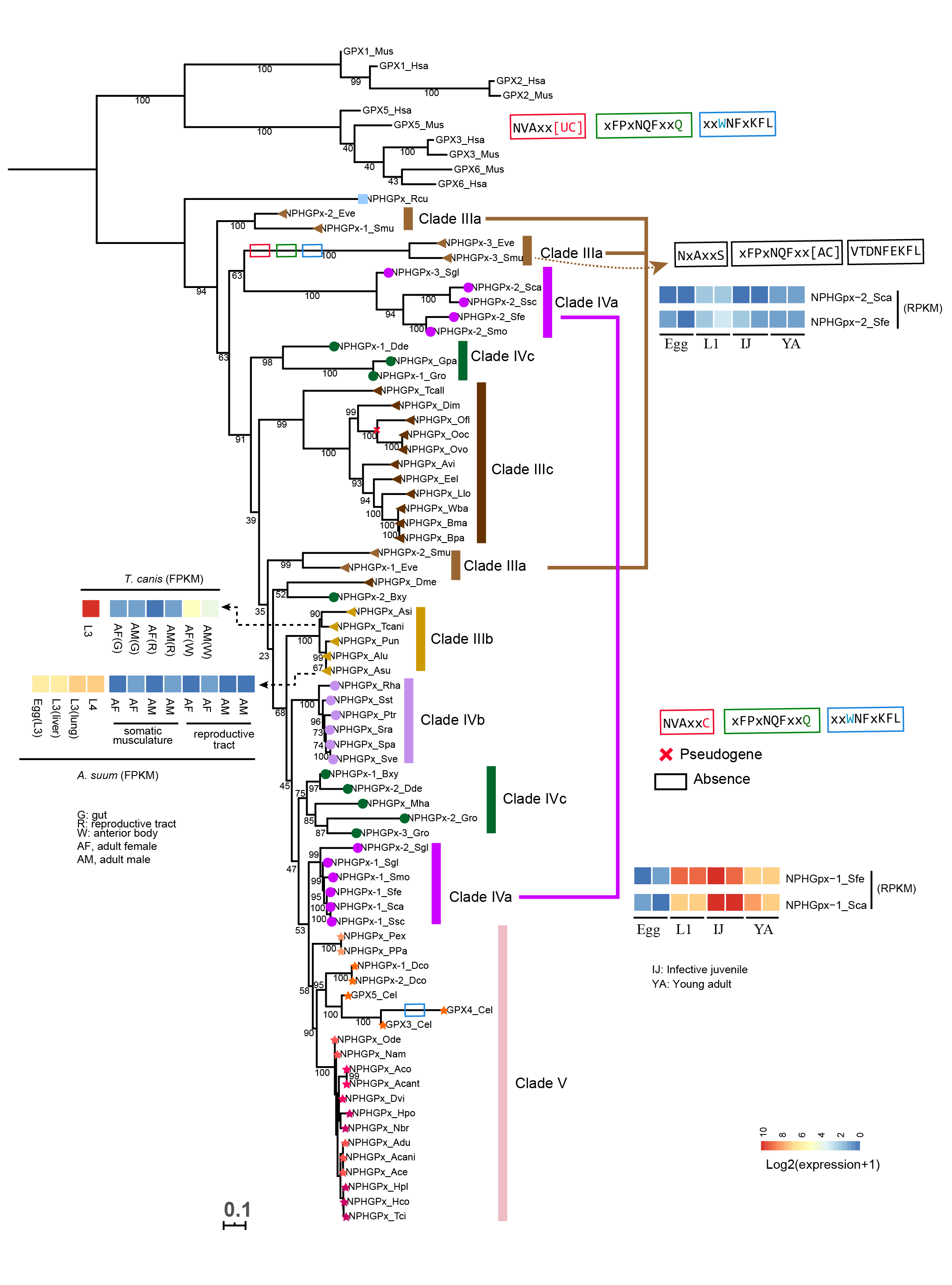
**

**Figure S16. The phylogeny of GPX group2 in nematodes, human and mouse.**

3 or 5 abbreviation for species, full name was shown in **Fig. 2** in the main text. The numbers at internal branches show bootstrap support values. The scale bar represents the number of amino acid substitutions per site.

_
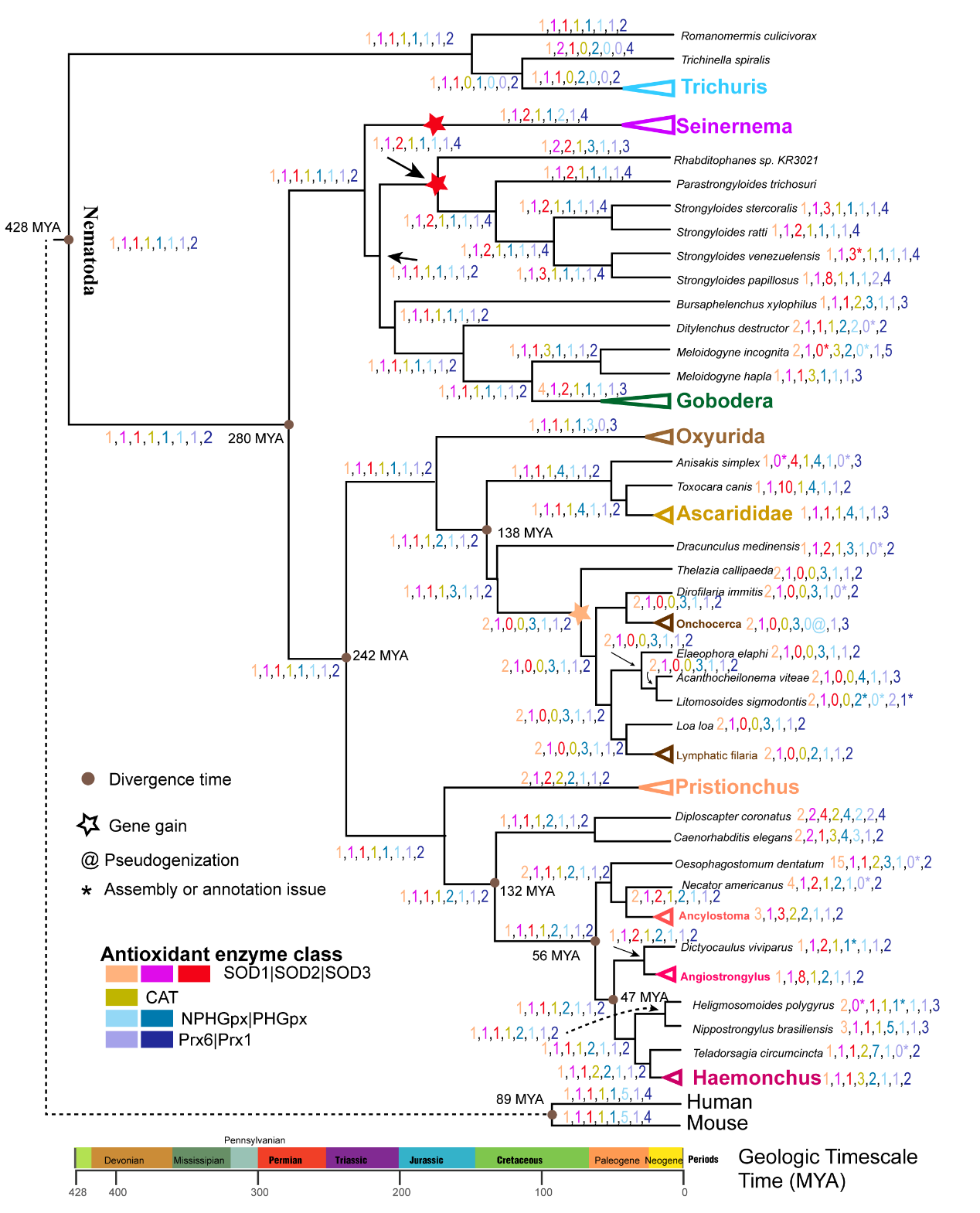
_

**Figure S17. Inferred antioxidant enzymes evolution in phylum Nematoda.** Numbers showed the inferred antioxidant enzyme number. Number color depicts different antioxidant enzymes. Only brown dot showed estimated divergence time retrieved from TimeTree database and our previous study^1^.

**Table S1. Scaffold/contig encoding Sod1 or Sod1-like information in six filarial nematode genomes**

| **Species Name** | **BioProject ID** | **Scaffold ID** | **Scaffold length (bp)** | **gene number in the scaffold** |  |
| --- | --- | --- | --- | --- | --- |
| *Acanthocheilonema viteae* | PRJEB4306 | nAv.1.0.scaf01318 | 17,274 | 3 | Sod1,Sod1-like |
| *Dirofilaria immitis* | PRJEB1797 | nDi.2.2.scaf01385 | 8,087 | 2 | Sod1 |
|  |  | nDi.2.2.scaf05833 | 1,026 | 1 | Sod1-like |
| *Onchocerca flexuosa* | PRJEB512 | OFLC_contig0006573 | 2,278 | 1 | Sod1-like |
|  |  | OFLC_contig0020995 | 920 | 1 | Sod1 |
| *Onchocerca ochengi* | PRJEB1809 | nOo.2.0.Scaf04000 | 6,074 | 2 | Sod1,Sod1-like |
| *Elaeophora elaphi* | PRJEB502 | EEL_contig0002910 | 2,337 | 1 | Sod1 |
|  |  | EEL_contig0001343 | 5,823 | 1 | Sod1-like |
| *Brugia pahangi* | PRJEB497 | BPAG_contig0002135 | 3,133 | 1 | Sod1 |
|  |  | BPAG_contig0005931 | 1,564 | 1 | Sod1 |
|  |  | BPAG_contig0003821 | 2,134 | 1 | Sod1-like |
|  |  | BPAG_contig0006192 | 1,511 | 1 | Sod1-like |

**Table S2. Summary of BUSCO results in seven root-knot nematode genomes.**


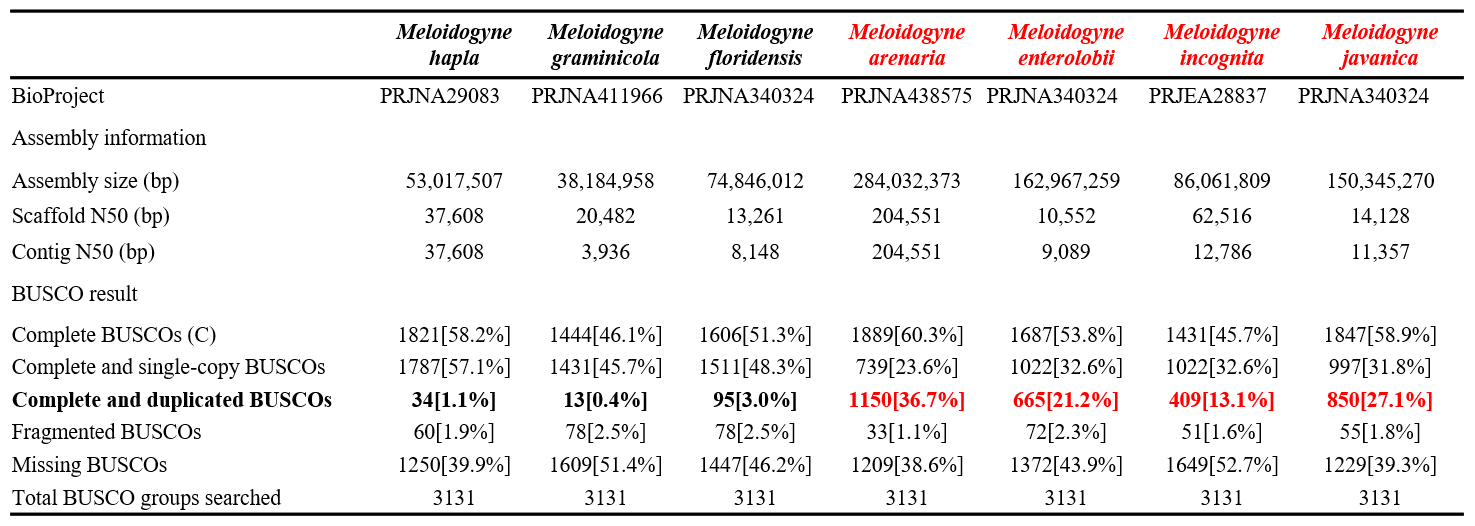


**Reference:**

1 Xu, L. *et al.* The genetic basis of adaptive evolution in parasitic environment from the *Angiostrongylus cantonensis* genome. *PLoS Negl Trop Dis* **13**, e0007846, doi:10.1371/journal.pntd.0007846 (2019).
